# Toward a Mechanistic Understanding of Reading Difficulties: Deviant Audiovisual Learning Dynamics and Network Connectivity in Children with Poor Reading Skills

**DOI:** 10.1523/JNEUROSCI.1119-24.2025

**Published:** 2025-02-27

**Authors:** Nada Frei, David Willinger, Patrick Haller, Gorka Fraga-González, Gustavo S.P. Pamplona, Amelie Haugg, Christina G. Lutz, Seline Coraj, Eva Hefti, Silvia Brem

**Affiliations:** ^1^Department of Child and Adolescent Psychiatry and Psychotherapy, University Hospital of Psychiatry Zurich, University of Zurich, Zurich 8032, Switzerland; ^2^Department of Psychology and Psychodynamics, Karl Landsteiner University of Health Sciences, Krems an der Donau 3500, Austria; ^3^Department of Computational Linguistics, University of Zurich, Zurich 8050, Switzerland; ^4^Neurolinguistics and Department of Psychology, University of Zurich, Zurich 8050, Switzerland; ^5^Family Larsson-Rosenquist Foundation Center for Neurodevelopment, Growth, and Nutrition of the Newborn. Department of Neonatology, University Hospital Zurich, University of Zurich, Zurich 8091, Switzerland; ^6^Neuroscience Center Zurich, University of Zurich and ETH Zurich, Zurich 8057, Switzerland; ^7^University Research Priority Program (URPP), Adaptive Brain Circuits in Development and Learning (AdaBD), University of Zurich, Zurich 8057, Switzerland

**Keywords:** associative audiovisual learning, children, computational modeling, dyslexia, effective connectivity by dynamic causal modeling, reinforcement learning drift-diffusion model

## Abstract

Mastering the associations between letters and their corresponding speech sounds (LSS) is pivotal in the early stages of reading development, requiring an effective reorganization of brain networks. Children with poor reading skills often show difficulties in LSS learning. To date, however, it remains unclear how the interaction of brain regions integral to the processing and integration of letters and speech sounds changes with LSS learning. Characterizing these changes and potential differences between children with typical (TR) or poor (PR) reading skills on both behavioral and neural levels is essential for a more comprehensive mechanistic understanding of reading impairments. In this study, we investigated brain network alterations underlying LSS learning and their association with reading skills using functional magnetic resonance imaging in 80 schoolchildren (6.9–10.8 years, 36 females, 27 PR) with a wide range of reading skills. We applied a reinforcement learning drift-diffusion model to LSS learning data and analyzed the corresponding effective connectivity and activation measures in the brain. While both groups learned well, PR showed slower adaptation of responses than TR as trials progressed. This could be explained by a slower adjustment of the drift rate and decision boundary while learning and longer nondecision times. Alongside deviant connectivity in the network of visual, auditory, and associative brain regions, PR also showed reduced striatal modulation of connectivity from visual to audiovisual association areas throughout learning. These findings indicate impaired information transfer to integrative areas, which aids to explain the difficulties in achieving proficient reading skills from a neuroscientific perspective.

## Significance Statement

This study investigated how children's brains learn to connect letters with speech sounds, a key step in reading development. Using brain imaging and computational models, we found that children with poor reading skills showed a less efficient adaptation of their decision-making strategy while learning letter–speech sound correspondences. The brain network connecting visual, auditory, and association areas in these children showed weaker connectivity and the modulation of connectivity by a key learning-related striatal region was diminished. These findings help explain the challenges faced by children with reading difficulties while learning to read and provide new insights into the brain mechanisms behind reading problems.

## Introduction

Reading is essential for participation in our text-mediated society and enables successful educational and professional development (Slavin et al., 2009). However, a considerable 5–10% of the population suffers from severe reading impairments, referred to as developmental dyslexia (Verhoeven et al., 2019). Current models suggest a multifactorial etiology of reading impairments (Peterson and Pennington, 2015; Catts and Petscher, 2022). One potential roadblock to attaining reading fluency may be alterations in letter–speech sound (LSS) representations that develop during reading acquisition (Blomert, 2011; Aravena et al., 2013; Hahn et al., 2014; Horbach et al., 2015; Altarelli et al., 2020). Previous studies reported deficient LSS learning and automation, weaker LSS representations, and subsequent difficulties in the application of learned associations in word reading and LSS matching tasks in children and adults with poor reading skills (Vaessen et al., 2009; Blau et al., 2010; Blomert, 2011; Richlan, 2014; Žarić et al., 2014; Fraga González et al., 2016; Aravena et al., 2018; Law et al., 2018; Pleisch et al., 2019; Verwimp et al., 2023). LSS learning performance is thus also considered as a potential early marker of subsequent reading outcomes in children (Horbach et al., 2015; 2018; Karipidis et al., 2018; Verwimp et al., 2023). Traditional performance parameters like accuracy and response times and isolated studies of regional brain activation, however, offer only limited insights into the mechanisms underlying LSS learning (Bonte and Brem, 2024). Yet, it remains unknown how brain regions of the reading network interact during associative learning and the establishment of LSS representations and whether deviations in this interaction affect the successful acquisition of fluent reading. A deeper understanding of the intricate processes underlying learning can be achieved by applying computational models, particularly when combining such models with neuroimaging data on brain network function (Huys, 2013). For instance, the reinforcement learning drift-diffusion model (RLDDM), amalgamating the reinforcement learning model (RLM; Sutton and Barto, 1998), and the drift-diffusion decision model (DDM; Ratcliff and Smith, 2004) have proven valuable in unveiling neural dynamics during learning and decision-making processes (Frank et al., 2015; Pedersen et al., 2017; Fontanesi et al., 2019; Miletić et al., 2020) and hold promise for gaining novel insights into associative learning of LSS correspondences. Specifically, prediction error (PE) signals play a dual role in associative learning, encoding surprise in the ventral striatum and sensory cortices and driving plasticity (den Ouden et al., 2009).

LSS learning entails cortical reorganization of the interaction of brain regions related to the processing and integration of auditory and visual information (van Atteveldt and Ansari, 2014; Dehaene et al., 2015; Hervais-Adelman et al., 2019; Pleisch et al., 2019). Deficient LSS learning in individuals with developmental dyslexia has been linked to deviant development and altered neural activity during cross-modal integration of LSS (Shum et al., 2011; Norton et al., 2015; Richlan, 2019). While a multitude of neurophysiological studies has pointed to alterations in terms of brain structure, function, and connectivity within and between regions of the reading network in individuals with reading impairments (Cao et al., 2008; Steinbrink et al., 2008; Rollins et al., 2009; van der Mark et al., 2011; Linkersdörfer et al., 2012; Richlan et al., 2013; Martin et al., 2016; Richlan, 2020; Yan et al., 2021; Devoto et al., 2022; Li et al., 2022; Di Pietro et al., 2023b), the current study seeks to enhance our understanding of potential alterations in brain mechanisms during LSS learning in children. Therefore, we first assessed LSS learning using an associative task, employing the RLDDM to discern behavioral variations in the learning process. Subsequently, we leveraged trial-wise learning parameters to analyze neural activation and connectivity data. This enabled us to pinpoint alterations linked to LSS learning within relevant brain networks. Finally, we examined discrepancies in brain network activation and connectivity during LSS learning among children with typical and poor reading skills.

## Materials and Methods

### Participants and group assignment

Our magnetic resonance imaging (MRI) recordings were part of a larger longitudinal intervention project, including behavioral assessments, electroencephalography, and MRI recordings. For the analyses of this article, the sample comprised all children whose data at the initial time point—prior to any intervention—met the quality criteria for both behavioral and functional MRI (fMRI) measures (see below). Ninety-nine (Swiss-) German-speaking children (age, 8.8 ± 1.5 years; age range, 7–10 years; 45 females; 90 right-handers) were recruited via advertisements and brochures distributed to local schools, speech therapists, pediatricians, and the local press. Exclusion criteria were MRI contraindications such as problems with lying still, claustrophobia or a general fear of the scanner, visual or auditory impairments, history of brain injury, current neurological or psychiatric disorders (all but dyslexia), or other major medical illnesses. Attention deficit hyperactivity disorder/attention deficit disorder (ADHD/ADD) was not an exclusion criterion, but participants were required to omit intake of medication for at least 24 h before the recordings and assessments (four children with poor and two with typical reading skills had a diagnosed ADHD/ADD). Nineteen children were excluded because their data did not meet our fMRI data quality criteria (*n* = 15) or because they made no errors during the associative LSS learning task (*n* = 4).

We thus considered a final sample of 80 children [age, 8.9 ± 0.8 years old; age range, 6.9–10.8 years; 44 females; 72 right-handers; Edinburgh Handedness Inventory (EHI); Oldfield, 1971] of a range of reading skills for the subsequent analyses. All children were in 1st to 3rd grade and had an estimated nonverbal IQ of ≥80 measured with the Reynolds Intellectual Assessment Scales (RIAS NX; Reynolds and Kamphaus, 2003; Brueggemann et al., 2006). Out of the 80 participants, 17 parents reported that their children are affected by developmental dyslexia. Of these, 11 children have a formal diagnosis of dyslexia, with four of these children diagnosed with comorbid ADHD. Here, it is important to note that the detection of dyslexia in the early school years (first to third grade) is not a primary focus in the Swiss school system. As a result, only the most severe cases tend to receive a formal diagnosis. Demographics are shown in Table 1. Children gave oral informed consent and their parents or legal guardians signed informed consent. We compensated all children via vouchers and small gifts for participation. The project was approved by the local ethics committee of the Canton of Zurich (BASEC No. 2018-01261) and neighboring Cantons in Switzerland.

**Table 1.** Descriptive statistics of cognitive assessments and task performance in the groups

	All (including IR)	TR	PR	PR versus TR	
	Mean/median ± SD			*t*(df)/*χ*2(df)	*p*
Participants	80	53	27		
Sex (female/male)	44/36	28/25	15/12	*χ*2(1) = 0.053	0.817
Handedness (right/left)	72/8	47/6	25/2	*χ*2(1) = 0.304	0.71
Age (years)	8.9 ± 0.8 [6.9, 10.8]	8.8 ± 0.7 [6.9, 10.3]	9.2 ± 0.7 [8.0, 10.8]	*t*_(78)_ = 2.11	0.038*
Nonverbal IQ^a^	105.0 ± 7.3 [88, 120]	105.1 ± 7.8 [88, 120]	104.6 ± 7.2 [90, 113]	*t*_(67)_ = −0.38	0.706
Working Memory (raw value)^b^	12.6 ± 2.4 [6, 19]	12.6 ± 2.6 [6, 18]	12.4 ± 2.0 [9, 19]	*t*_(78)_ = −0.28	0.78
**Reading-related skills**					
Word and Pseudoword reading fluency (perc.)^c^	35.1 ± 29.8 [1, 99]	50.4 ± 24.9 [16.5, 99]	5.0 ± 4.3 [1, 14.5]	*t*_(78)_ = −9.28	<0.0001****
Word reading fluency (perc.)^d^	40.4 ± 24.3 [4, 100]	48.9 ± 24.8 [4, 100]	23.7 ± 9.9 [5, 39]	*t*_(78)_ = −5.02	<0.0001****
Sentence reading (quot.)^e^	87.6 ± 19.5 [62, 138]	96.2 ± 18.0 [63, 138]	71.2 ± 8.3 [62, 88.5]	*t*_(77)_ = −6.80	<0.0001****
Spelling (*t*-score)^f^	47.8 ± 12.0 [26, 76]	50.3 ± 12.7 [26, 76]	42.8 ± 8.4 [28, 64]	*t*_(77)_ = −2.75	0.007**
Reading comprehension (*t*-score)^g^	45.9 ± 11.6 [25, 75]	50.8 ± 10.2 [33, 75]	36.8 ± 8.0 [25, 58]	*t*_(74)_ = −6.14	<0.0001****
RAN mean animals, objects (raw value)^h^	0.74 ± 0.17 [0.39, 1.13]	0.79 ± 0.16 [0.45, 1.13]	0.65 ± 0.15 [0.39, 0.93]	*t*_(77)_ = −3.87	<0.0001****
**In-scanner associative learning task performance**					
Percentage of hits	78.3 ± 10.8	78.3 ± 11.1	78.3 ± 10.0	*t*_(78)_ = 0.013	0.99
Percentage of errors	16.9 ± 10.1	16.9 ± 10.0	16.4 ± 8. 8	*t*_(67)_ = −0.198	0.844
Percentage of missed responses	4.1 ± 3.9	4.1 ± 3.8	5.3 ± 4.4	*t*_(67)_ = 1.156	0.252
Response time correct (median, ms)	1,434 ± 143	1,423 ± 145	1,458 ± 132	*t*_(78)_ = 1.656	0.102
Response time incorrect (median, ms)	1,502 ± 205	1,464 ± 218	1,543 ± 170	*t*_(78)_ = 0.942	0.349
**Modeling parameters**					
Decision boundary (*a*)	3.17 ± 0.13	3.18 ± 0.13	3.14 ± 0.11	*t*_(78)_ = −1.12	0.22
Nondecision time (*t*)	0.73 ± 0.13	0.70 ± 0.13	0.77 ± 0.10	*t*_(78)_ = 2.44	0.017*
Drift rate modifier (*v*_mod_)	0.97 ± 0.32	0.98 ± 0.32	0.94 ± 0.28	*t*_(78)_ = −0.55	0.582
Learning parameter (*η*)	0.19 ± 0.002	0.19 ± 0.002	0.19 ± 0.002	*t*_(78)_ = 1.08	0.285
Starting point (*z*)	0.75 ± 0.01	0.75 ± 0.01	0.75 ± 0.01	*t*_(78)_ = 1.81	0.241

Note: TR, children with typical reading skills; PR, children with poor reading skills; IR, children with intermediate reading skills; SD, standard deviation; *, significant *p* values; [],range; response time measures are reported as median, all other measures as mean.

a IQ measured with Reynolds Intellectual Assessment Scales (RIAS NX).

b Working memory using subtests from the Wechsler Intelligence Scale for Children (WISC-IV digit span forward and backwards: total raw value).

c Salzburger Lese- und Rechtschreibtest (SLRT-II) number of correctly read items per minute (mean of percentiles of words and pseudoword reading).

d SLRT-II number of correct items (words) within 1 min (raw values).

e Covert sentence reading speed using the Salzburger Lese-Screening (SLS (1–4, 2–9); reading quotient.

f Spelling performance using SchreibOn *t*-score.

g Reading comprehension using the Leseverständnistest für Erst- bis Siebtklässler (ELFE II) *t*-score.

h Rapid automatized naming test (RAN): number of correct items per second [mean of RAN objects (short items with 1 syllable) and RAN animals names (long items with 3 syllables) item/sec]. *p < 0.05, **p < 0.01, ***p < 0.001, ****p < 0.0001.

The sample was further divided into two groups based on the children's mean reading score from a standardized reading and spelling test battery, the “Salzburger Lese- und Rechtschreibtest” (SLRT-II; Moll and Landerl, 2010; see below). This reading score, derived from children's performance on word and pseudoword reading fluency subtests, was selected to directly assess foundational reading skills rather than spelling or higher-level reading comprehension skills, while ensuring consistency with our previous research (Haugg et al., 2023; Di Pietro et al., 2023a,b). Children with typical reading skills scored at or above the 16th percentile (TR ≥16), while children with poor reading skills scored below the 16th percentile (PR <16). The 16th percentile, corresponding to a standard score of one standard deviation below the mean, is commonly used to identify significantly below-average performance in educational and psychological assessments, such as dyslexia screenings. This resulted in groups of 27 children with poor reading skills (PR) and 53 children with typical reading skills (TR) for the core analyses of the main text. Using the 1 SD cutoffs furthermore improves the comparability of our results with those from similar research in our group (Haugg et al., 2023; Di Pietro et al., 2023a,b) and also others (Hannigan et al., 2015; Moll et al., 2016; Vanvooren et al., 2017; Law et al., 2018; Banfi et al., 2019; Dębska et al., 2021; Yan et al., 2024). For extended data analyses, we additionally excluded 11 children with reading skills in the 16–25th percentile range to ensure a clearer distinction between intermediate to strong (ISR, *n* = 42) and poor readers (PR, *n* = 27). While no specific a priori power analysis was conducted for the analyses of this study, the sample size is expected to be adequate for model fitting and establishing brain–behavior correlations based on previous research (Diwadkar et al., 2012; Morken et al., 2017; Di Pietro et al., 2023b). However, we acknowledge recent findings suggesting that current standards in psychological studies may be underpowered, indicating potential for improvement in future research (Szucs and Ioannidis, 2017).

### Cognitive assessments

The behavioral assessment battery was administered prior to the MRI recording and included nonverbal IQ, working memory using subtests of the Wechsler Intelligence Scale for Children (WISC-IV digit span forward and backward, (Baron, 2005; Wechsler, 2011), and overt reading fluency (word reading) and decoding (pseudoword reading) using the SLRT-II (Moll and Landerl, 2010). Furthermore, we assessed the speed of covert sentence reading using the Salzburger Lese-Screening [SLS (1–4, 2–9); Mayringer and Wimmer, 2003], spelling performance using SchreibOn (May, 2013), sentence reading comprehension using the “Leseverständnistest für Erst- bis Siebtklässler“ (ELFE II; Lenhard and Schneider, 2006), and speed of naming animals and objects using two versions of the Rapid Automatized Naming with either short (1 syllable) or long (3 syllables) items (RAN; Denckla and Rudel, 1976; Gordon et al., 2021).

### Experimental design and data

#### Audiovisual associative learning task design

**Figure 1.**
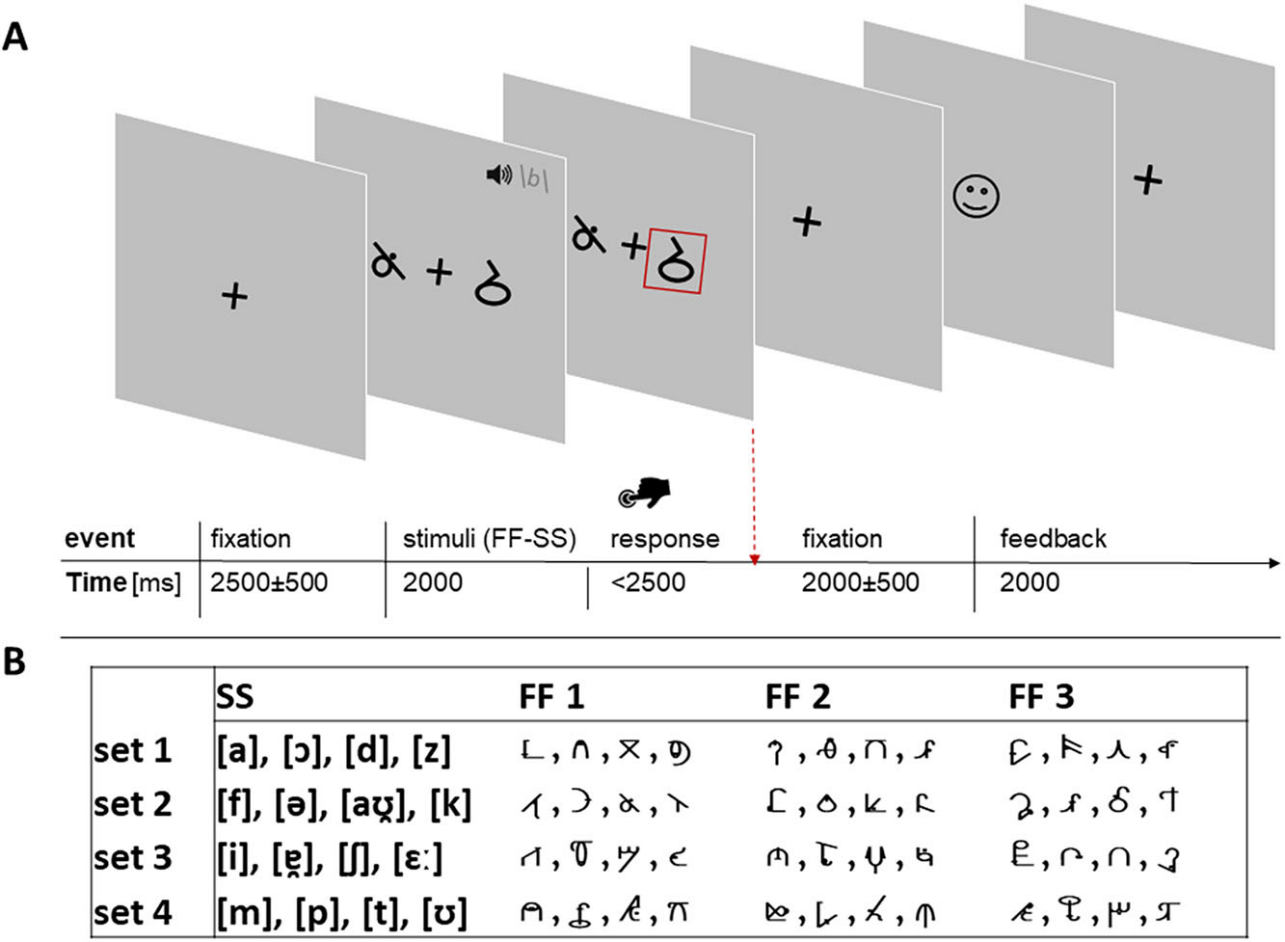
***A***, Experimental design for the associative learning task**.** Two runs consisted of four FF–SS associations with 40 trials each. In each trial, one SS and two FFs were presented for 2,000 ms. Visual feedback for correct or incorrect responses was presented via a happy or unhappy face emoji for 2,000 ms. ***B***, Possible FF–SS combinations used in the associative learning task. SS are represented in the International Phonetic Alphabet and shown here in phonetic notation. They were presented together with one of the possible FF combinations. Three FF groups of four characters (FF 1, FF 2, FF 3) were used to build different versions of the FF–SS combinations. Each participant was randomly assigned to two sets of SS–FF pairs performed in different runs. The sets served as backups in case excessive motion occurred during one of the first two runs, allowing participants to repeat the task.

Children who already can read can be trained to learn novel associations between unfamiliar, nonstandard font characters and speech sounds, simulating LSS learning (Brem et al., 2010; Aravena et al., 2013; Fraga González et al., 2015; Aravena et al., 2018; Karipidis et al., 2018; Verwimp et al., 2023). The learning of such false font character (FF)–speech sound (SS) associations was conducted during fMRI acquisition. The task consisted of two 6 min runs of 40 trials each. Four FF–SS associations per run had to be learned deterministically during 10 repetitions. In each trial, children received visual stimulation of two FFs in black in the middle of a gray background using video goggles (VisuaStim Digital; Resonance Technology) with a resolution of 800 × 600 pixels for 2,000 ms using Presentation (v 16.4, www.neurobs.com). Simultaneously, children were presented with a binaural auditory German SS through headphones (MR Confon; Fig. 1*A*). Children were asked to select the FF corresponding to the SS. They received feedback to learn the correct associations. The two FFs were presented to the right and left of a fixation cross in the center of a gray screen. Children were instructed to press the corresponding button on a two-button response pad (Cambridge Research Systems) with their index and middle fingers to indicate their choice as soon as they recognized the matching pair. Visual feedback signaling correct or incorrect responses via a happy or unhappy emoji were provided at the end of each trial for 2,000 ms. A fixation cross of randomly varying duration was presented for 2,500 ± 500 ms before stimulus and for 2,000 ± 500 ms before feedback presentation (Fig. 1*A*). The task was implemented using Presentation (v 18.0; Neurobehavioral Systems; www.neurobs.com).

The FF stimuli were selected from the lowercase symbols of the Brussels Artificial Character Sets (BACS), a pool of artificial characters that are similar to letters in terms of their visual complexity (Vidal et al., 2017). In each run, participants had to learn four different sets of FF–SS associations. The four sets of FF–SS associations, each with three possible corresponding FF versions, are illustrated in Figure 1*B*. These three different FF groups of four characters (FF 1, FF 2, FF 3) were combined with the four SS possibilities, and the resulting FF–SS stimulus sets were randomly assigned to participants. Each participant performed two different sets in each run. The SS stimuli were recorded by a female, native German speaker. All audio files had a sampling rate of 44.1 kHz and 16 bit rate. The files were normalized using the *normalize* function of Audacity (v2.3.0; www.audacityteam.org).

#### Procedure

We initially conducted training sessions for all children in a mock scanner to help them acclimate to the MRI environment and scanning procedures prior to the actual MR recording. Then, children received instructions and completed a practice run outside of the scanner (∼2 min) to familiarize themselves with the task. In the scanner, they did another short practice run immediately before the task. We further conducted an audio test to ensure that they could properly hear the speech sounds. After each run, functional data was analyzed for excessive motion using the realignment analysis step of the Statistical Parametric Mapping toolbox (SPM12 version 7487; Wellcome Trust Centre for Neuroimaging, University College London; http://www.fil.ion.ucl.ac.uk/spm) running on MATLAB (version R2019a). If excessive motion distorted the data quality, the run was repeated with a new FF–SS set to obtain two runs of high data quality. There was no difference between children with typical and poor reading skills in the number of children who had to repeat runs of the learning task. Among the 27 children with poor reading skills, nine needed to repeat one or two runs. In the group of 53 children with typical reading skills, 18 required repetition runs. A chi-square test revealed no significant association between reading skill group and the need for run repetition (*χ*^2^_(1)_ = 0.05, *p* = 0.823). A linear mixed model (LMM) analysis of motion (framewise displacement) during fMRI runs showed no significant group-by-bin interaction (*t*_(481.0)_ = −1.43, *p* = 0.152). However, there were main effects of group (*t*_(153.5)_ = 2.34, *p* = 0.020) and bin (*t*_(481.0)_ = 2.60, *p* = 0.010).

#### MR acquisition and preprocessing

MRI data was recorded at the Psychiatric Hospital of the University of Zurich using an Achieva 3 Tesla scanner (Philips Medical Systems) equipped with a 32-channel receive head coil. Functional images of the associative learning task were acquired using T2*-weighted whole-brain images with multiband echo-planar pulse sequence [273 volumes; repetition time (TR), 1.33 s; echo time (TE), 35 ms; flip angle, 80°; field-of-view (FOV), 192 × 192 mm; acquisition matrix, 64 × 64; gap, 0.299 mm; 42 slices; isotropic voxel size, 3.0 mm^3^; matrix size, 64 × 62 px; multiband factor, 2; SENSE acceleration factor, 2; SofTone factor, 2]. High-resolution T1-weighted anatomical images were acquired for each participant and recorded using a magnetization-prepared rapid acquisition gradient echo (MPRAGE) sequence with the following parameters: TR, 6.8 s; TE, 3.2 s; aligned at the anterior-posterior commissure plane; flip angle, 9°; isotropic voxel size, 1.0 mm^3^; field-of-view, 270 × 255 mm^2^; number of slices, 176.

Data were preprocessed and statistically analyzed with the toolbox SPM12 (scripts publicly available https://osf.io/g8rjx/). Data preprocessing consisted of slice-time correction, realignment, segmentation, and coregistration. To normalize our functional images, we created a customized pediatric anatomical template (mean age, 8.83 years; range, 6.9–10.8 years) using the Template-O-Matic toolbox (TOM; Wilke et al., 2008). Next, we smoothed the data with an 8 mm full-width at half-maximum Gaussian kernel and resampled them to isometric 3 × 3 × 3 mm^3^ 386 voxels. Finally, volumes exceeding the scan-to-scan motion of 1.5 mm were repaired using linear interpolation between the nearest unrepaired scans as implemented in the ArtRepair toolbox (Mazaika et al., 2007). We also flagged unrepaired volumes surrounded by volumes with excessive movement. Datasets containing >10% of repaired or flagged volumes were not considered for further analysis. Accordingly, the data of 15 children out of 99 children who did not meet the stringent quality criteria in one or both runs were excluded. Among those 15 children excluded due to fMRI data quality issues, 9 were children with typical reading skills (6 had only one usable run, and 3 had none), and 6 were children with poor reading skills (5 had only one usable run, and 1 had none). Additionally, three children with typical reading skills and one with poor reading skills were excluded due to task performance issues (see details below).

### Statistical analyses

#### Performance during associative learning task

To assess the overall performance in the FF–SS associative learning task, we computed the accuracy, defined as the proportion of correct answers for a given pair, and the response time (RT) of correct trials. The maximally allowed RT for the children was 2,500 ms. RTs above 2,500 ms were defined as missed answers and treated separately in the model. We divided each of the two FF–SS runs into four bins of equal length (10 trials) to examine the learning progress within runs. If the number of errors per run was zero, participants were excluded. From the sample of 84 children with adequate fMRI data quality, four children had to be excluded from modeling analyses, resulting in a final sample of 80 children.

We employed an LMM to analyze the data. The dependent variables RT and accuracy were predicted by a model incorporating both fixed and random effects. The fixed effects included the interaction between time bin and reading status, as well as the covariate age. The random effects structure accounted for the nested design, with observations nested within participant and session. Specifically, the model allowed for random intercepts for each participant within each session, capturing the variability in RT and accuracy across them. For post hoc comparisons, the bin variable, originally continuous, was converted to a factor to assess its categorical impact on the dependent variable. This approach facilitated the examination of within-subject changes in the response variables across different levels of bin, while controlling for reading ability and age. Where applicable, degrees of freedom in the LMMs were estimated using Satterthwaite's method.

In the extended data (Extended Data Table 3-1) and the supplemental material available in the public repository (https://osf.io/g8rjx/), we also report the additional dimensional LMM analysis for RT, accuracy, drift rate (the average speed at which evidence is gathered to reach a decision; see below, Reinforcement learning drift-diffusion model), and decision boundary (represents the critical threshold of evidence required to commit to a choice; see below, Reinforcement learning drift-diffusion model) including children's reading scores as a continuous covariate of interest and LMM analyses excluding the 11 children with reading skills in the 16th–25th percentile range (intermediate readers IR). The significance level for all statistical tests was *p* < 0.05, two-tailed.

We performed these analyses in R (R Core Team, 2023; R: A language and environment for statistical computing. R Foundation for Statistical Computing; available from: https://www.*R*-project.org/). Analyses scripts publicly are available at https://osf.io/g8rjx/.

#### Reinforcement learning drift-diffusion model

To gain more detailed insights into learning and decision-making processes, we employed a reinforcement learning drift-diffusion model (RLDDM; Pedersen et al., 2017) to the performance data of our fMRI FF–SS learning task. We implemented the RLDDM in the context of FF–SS learning in a hierarchical Bayesian inference approach [as described in Pedersen et al. (2017) and applied in Fontanesi et al. (2019); Fraga González et al. (2025)]. The framework of RLDDM integrates the DDM (Ratcliff and Smith, 2004), a sequential sampling model depicting decision-making as evidence accumulation over time, with principles of the RLM, which describes how learning is shaped by feedback (Sutton and Barto, 1998). This is done by estimating the drift rate from the DDM from the associative strength (AS) from RLM (compare below). AS refers to the agent's learned association between a stimulus pair that is updated by the prediction error (PE) of the previous occurrence. The PE is the difference between the observed outcome and the AS. The drift rate represents the average speed to accumulate evidence to decide on one alternative. Drift rate is estimated trial-wise as the average AS of the two presented stimuli. It is positive in trials when a correct response is given and negative when an incorrect response is given. A scaling factor drift rate modifier (*v*_mod_) multiplies the difference in expected rewards, which results in the drift rate (variable *v̂*) in each trial, i.e., *v̂* = *v*_mod_ * ((AS_correct pair_ + AS_incorrect pair_)/2).

AS and learning rate (*η*) are variables of the RLM and drift rate, decision boundary (*a*), nondecision time (*τ*), and starting point (*z*) variables of the DDM. The learning rate embodies the step size of the update, and the decision boundary in RLDDM is the threshold that determines how much evidence is needed before making a choice between alternatives. The decision boundary accounts for any speed–accuracy trade-off. A higher decision boundary is usually related to slower and more accurate responses and vice versa. While nondecision time disentangles the component of the RT that can be attributed to lower-level processes rather than decision-making, such as stimulus encoding and motor processes, the starting point refers to the beginning of the evidence accumulation.

**Figure 2.**
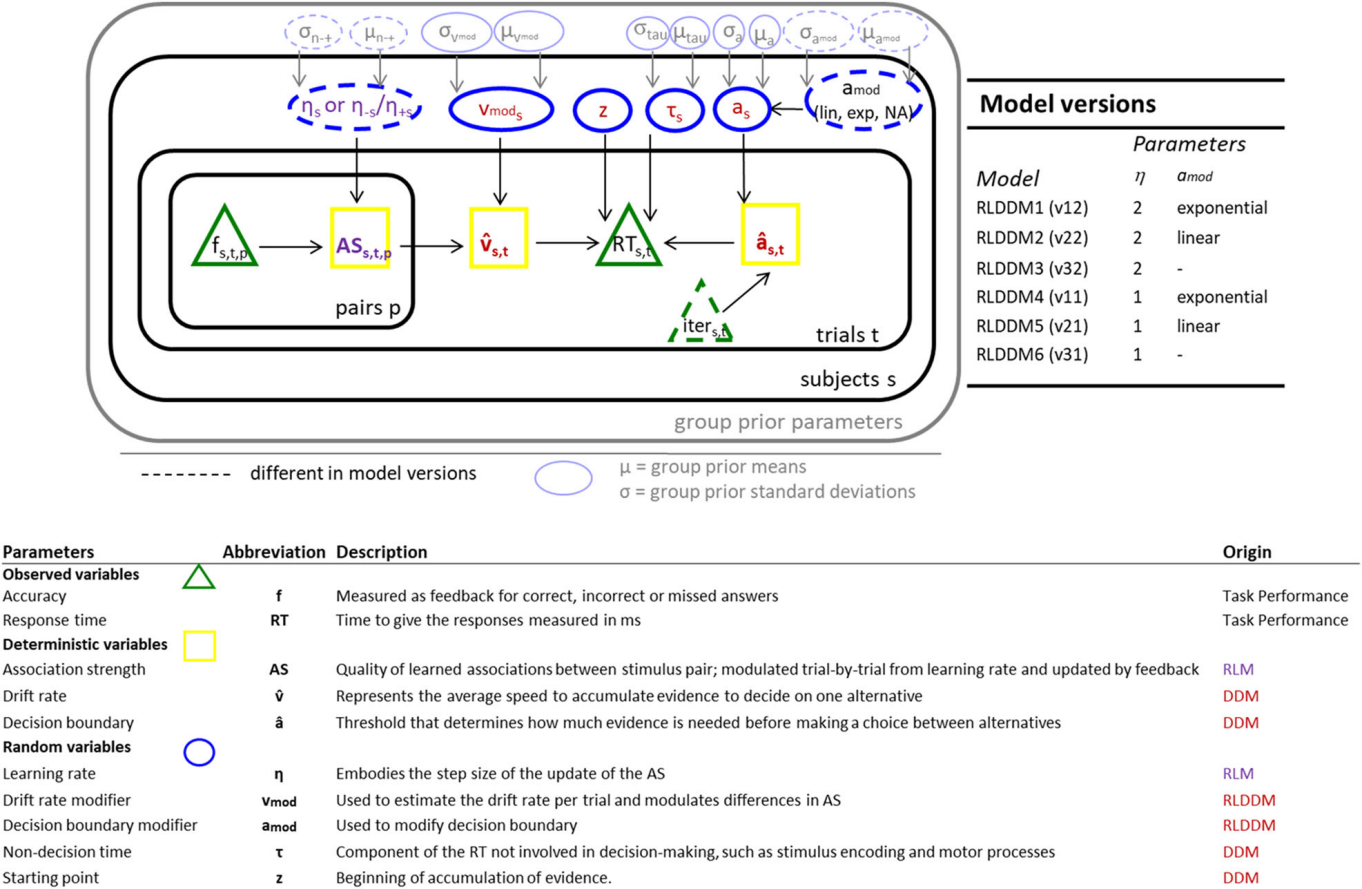
Graphical representation of the Bayesian hierarchical RLDDM. Deterministic, observed, and random variables are labeled in yellow squares, green triangles, and blue ovals, respectively. The model-free parameter *v*_mod_ was used to estimate the drift rate per trial and modulated differences in association strength (AS). AS was also modulated trial-by-trial from the learning rate(s) (*η*_s_ or *η*_−s_/*η*_+s_) and updated by feedback. We estimated the parameters in a hierarchical Bayesian framework. We estimated subject parameters based on group means *μ* and standard deviation *σ*. Dashed lines represent variants for model versions (see summary on the right). RLDDM 1–3 include two separate learning rates for negative and positive PE. RLDDM 3 and 6 have a static decision boundary, RLDDM 1, 2, 4, and 5 include trial-wise modified decision boundary, while in RLDDM 1 and 4 the mapping between *a*_mod_ and *â* was exponential, in RLDDM 2 and 5 it is linear. For the Markov chain Monte Carlo traces for key group parameters, see Extended Data Figure 2-1.

The model parameters can be categorized into three deterministic variables: the AS, the drift rate, and the decision boundary. Next latent variables to be estimated are as follows: the learning rate, the drift rate modifier (*v*_mod_), the decision boundary modifier (*a*_mod_), nondecision time, and the bias of the starting point (*z*). The model outputs two observed variables, the RT (continuous) and the feedback (binary), which is a measure of accuracy. Accuracy and RT distributions depend on several decision parameters, such as nondecision time, starting point, and decision boundary (compare Fig. 2).

10.1523/JNEUROSCI.1119-24.2025.f2-1 Figure 2-1 **Traces of Markov chains for the group parameters.** This figure displays the Markov chain Monte Carlo (MCMC) traces for key group parameters in our drift diffusion model analysis. Each panel represents a different parameter, showing how the estimates evolve over the course of the MCMC sampling process. Each trace represents 5’000 retained post-warmup samples from four independent chains. Download Figure 2-1, TIF file.

We explored two model variations: one with distinct learning rates for positive and negative prediction errors and another with a unified learning rate. This approach allowed us to investigate the potential asymmetry in learning from different feedback valences and to compare the insights gained against those from a model-free perspective, which lacks the dynamic representation of evidence accumulation inherent to the RLDDM. Additionally, we explored in those models various implementations of the decision boundary parameter, which could either remain static or dynamically adapt across trials according to a linear or power function to investigate how changes in the speed–accuracy trade-off manifest throughout the course of a learning task (refer to the model fitting details below).

Finally, we assessed brain responses modulated by model-derived parameters of the best model fit, specifically the PE and AS to determine how the reading network is involved in associative learning.

### Model implementation and fitting

To find the best fitting model, we compared six different models that differed with respect to the learning rate parameter: they included either one single learning rate for correct and incorrect trials or separated learning rates by trial *η*_+_ and *η*_−_. The latter was used to update the AS after positive and negative PEs (Pedersen et al., 2017). Three of the RLDDM versions (RLDDM 1–3) include two separate learning rates for negative and positive PE. RLDDM 3 and 6 have a static decision boundary, while the other models (RLDDM 1, 2, 4, 5) include trial-wise modified decision boundaries. While in RLDDM 1 and 4, the mapping between *a*_mod_ was exponential, in RLDDM 2 and 5 it is linear (Table 2, Fig. 2).

**Table 2.** Results and comparison of all models

	Parameters		Trial-wise predictive accuracy		
Model	*η*	*a*_mod_	*p*_waic_	WAIC	Rank
RLDDM1	2	Exponential	203.9	11,112.4	2
RLDDM2	2	Linear	202.7	11,137.7	4
RLDDM3	2	-	200.4	11,126.2	6
**RLDDM4**	**1**	**Exponential**	168.1	**10,472**.**0**	**1**
RLDDM5	1	Linear	167.1	10,525.6	3
RLDDM6	1	-	163.4	10,510.4	5

Rank 1 RLDDM4 is the best fit, highlighted in bold; indicated by the lowest WAIC, Widely Applicable Information Criterion; *p*_WAIC_ represents the effective number of parameters.

We implemented and fitted the RLDDM model on a single group of participants in Rstan (Rstan Development Team, 2016; https://mc-sstan.org/) a state-of-the-art platform for statistical modeling using the command line interface cmdstan in R (R Core Development Team 2013). To estimate the posterior, we used Stan's default Markov chain Monte Carlo algorithm (Duane et al., 1987; Neal, 2011). We simultaneously estimated full posterior distributions of the individual and group parameters—enabling mutual constraining and conveying uncertainty associated with parameter estimates. RLDDM assumes data to be distributed according to the Wiener first-passage time-distribution (WFPT): RT*_i_*_,*t*_ ∼ WFPT (Θ = {*a*, *τ*, *z*, *v_t_*}). We ran the models with four parallel chains with 10,000 iterations each retaining every second sample to mitigate autocorrelation, including 4,000 warm-ups. We assessed the convergence of Markov chains using visual inspection (Extended Data Fig. 2-1) and the criterion of the diagnostic $\hat{R}$ < 1.01, which was satisfied by the parameters. Modeling scripts are publicly available at https://osf.io/g8rjx/.

### Model comparison

We compared model variants to determine which model described the best relative fit. As a metric for model comparison, we computed the Widely Applicable Information Criterion (WAIC; Watanabe et al., (2013)). The *p*_WAIC_ represents the effective number of parameters. Having the lowest WAIC suggests that the model's complexity is compensated by its superior fit to the data.

The model comparison showed that the model (RLDDM4) with a single learning rate and the exponential decision boundary (*a*_mod_) fitted the response data best. Therefore, the RLDDM4 with a single learning parameter and exponential decision boundary provided the best description of learning, choices, and response time (Table 2) in our children's data.

#### RLDDM parameters

From the model that best fitted the data (RLDDM4), we extracted the subject-specific parameters nondecision time (*τ*), starting point (*z*), learning rate (*η*), the trial-wise parameters decision boundary (*a*), and drift rate (*v̂*). To examine group differences, we conducted two-sample *t* tests with subject-specific parameters, as well as trial-wise parameters averaged across trials. Further, we also conducted LMMs with the extracted trial-wise parameters. The dependent parameters drift rate and the decision boundary were predicted by a separate model incorporating both fixed and random effects. The fixed effects included the interaction between bin and reading status, as well as the covariate age. The random effect structure accounted for the nested design, with observations nested within participants and sessions. Specifically, the model allowed for random intercepts for each participant within each session, capturing the variability in drift rate and decision boundary. For post hoc comparisons, the bin variable, originally continuous, was converted to a categorical factor to assess its association with the dependent variable. This approach facilitated the examination of within-subject changes in the response variables across different levels of bin, while controlling for reading ability and age. Where applicable, degrees of freedom in the LMM were estimated using Satterthwaite's method. Additionally, we also ran Bayesian *t* tests and Bayesian LMM to examine group differences and the associations of bin and reading status with drift rate and the decision boundary, preserving the parameter uncertainty within participants.

We performed these analyses in R (R Core Team, 2023; R: A language and environment for statistical computing. R Foundation for Statistical Computing; available from https://www.R-project.org/).

#### Whole-brain fMRI analysis

##### First-level analyses

We built two general linear models (GLMs). Both GLMs included two vectors of interest (stimulus onsets and feedback onsets) convolved with the canonical hemodynamic response function as implemented in SPM12. In addition to the six realignment parameters, a vector with the flagged scans whenever available and one vector with missed trials whenever available were included as regressors of no-interest in these models. Subsequently, we incorporated model parameters obtained from the best fitting model (RLDDM4) as parametric modulators in a GLM. Specifically, in this RLDDM4, AS was included as the parametric modulator of the stimulus onsets, while the prediction error (PE) served as the parametric modulator of the feedback onsets.

##### Second-level analyses

We used one-sample *t* tests to examine overall stimulus and feedback processing on the whole-brain level and how the model parameters modulated brain activity at stimulus onset (AS or drift rate) and feedback onset (PE). Because of the high sensitivity of this analysis, we applied a stringent correction using an initial FWE-corrected cluster-defining threshold (*p*_FWE_) of 0.05 and a FWE-corrected cluster-level threshold (*p*_FWEc_) of 0.05 to identify and display the core clusters of our findings (Fig. 4 and tables of the *t-*maps in the Extended Data 4-1, 4-4, 4-5).

Two-sample *t* tests were then used to analyse the difference between poor and typical readers in contrast (i.e., beta) maps during stimulus or feedback presentation. For these analyses we employed an initial cluster-defining threshold *p*_CDT_ of 0.001 and a FWE-corrected cluster-level threshold (*p*_FWEc_) of 0.05 to present our findings (Extended Data Fig. 4-2, Table 4-2). Anatomical labels for the resulting brain regions were obtained using the SPM Anatomy Toolbox (Eickhoff et al., 2013). All GLMs are publicly available at https://osf.io/g8rjx/.

#### Effective connectivity analysis using dynamic causal modeling

We used dynamic causal model (DCM) as implemented in SPM12 to investigate effective connectivity between the LSS network and differences between typical and poor readers. To this end, we defined a left-hemispheric network of regions relevant to audiovisual learning of letter–speech sound correspondences based on previous reports (Werner and Noppeney, 2010; Aravena et al., 2018; Li et al., 2019; Xi et al., 2019; Yan et al., 2023), as well as converging with the network activated in our task (GLM 1; Extended Data Table 4-1). This network included four regions of interests (ROIs): an audiovisual integration region in the left superior temporal sulcus (STS) [*x* = −65, *y* = −24, *z* = 9 (mm MNI); Calin-Jageman and Cumming, 2019; Li et al., 2019], the left primary auditory cortex [PAC: *x* = −47, *y* = −21, *z* = 6 (mm MNI)] as the auditory input area, the left ventral occipitotemporal cortex as visual orthographic processing region [vOT: *x* = −41, *y* = −66, *z* = −12 (mm MNI); Liebig et al., 2017], and the bilateral putamen (PUT; Table 3), which supports the creation and retrieval of new associations during the learning task (den Ouden et al., 2009). For all four ROIs, we created individual spheres of a 6 mm radius centered on the activation maximum of each participant within the given search region. We used a spherical volume with *r* = 12 mm as a search region for the three functionally defined regions, and a bilateral anatomical mask was retrieved from the Harvard-Oxford atlas for the anatomically defined putamen. Subsequently, we extracted the first eigenvariate of the time course of active voxels (*p* < 0.05, uncorrected), whereas the contribution of motion parameters, volumes with excessive head movement, and effects of run were regressed out. Out of the 80 children with available data, the data of five typical readers did not show significant voxels in the PUT and were thus excluded, leaving 75 children for the effective connectivity analysis (48 TR, 27 PR).

**Table 3.** ROIs for DCM analysis

	MNI coordinates (mm)				
Hemisphere	Brain region	*x*	*y*	*z*	Sphere radius (mm)
Left	vOT	−41	−66	−12	6
Left	PAC	−47	−21	6	6
Left	STS	−65	−24	9	6
Bilateral	PUT	−26	3	1	6

vOT, ventral occipitotemporal cortex; PAC, primary auditory cortex; STS, superior temporal sulcus; PUT, putamen.

The first-level DCM included one driving input (audiovisual stimulus) that entered the visual letter (vOT) and auditory (PAC) regions and a second driving input (associative strength) that entered the putamen. The base model consisted of bidirectional connections between the three cortical regions. We allowed the putamen to exert modulatory influence on those bidirectional connections, thereby constructing a nonlinear DCM (Stephan et al., 2008). The first-level DCMs were estimated using an empirical Bayesian inversion scheme, and group inference on model structure and connectivity strengths was performed using the Parametric Empirical Bayes framework (Friston et al., 2015). We analyzed intrinsic connections (*A*-matrix) and modulations (*D*-matrix) separately for differences between typical and poor readers including sex, age, and handedness as covariates. We employed a Bayesian model reduction procedure that pruned away any model parameter that did not contribute to the model evidence (Friston et al., 2016). For the remaining parameters, we calculated their averages across nested models weighted by the respective posterior probability. The significance threshold for a parameter was a 95% posterior probability of being present versus absent based on the model evidence. Scripts are publicly available at https://osf.io/g8rjx/.

## Results

### Task performance during LSS learning

#### Accuracy and response time

**Figure 3.**
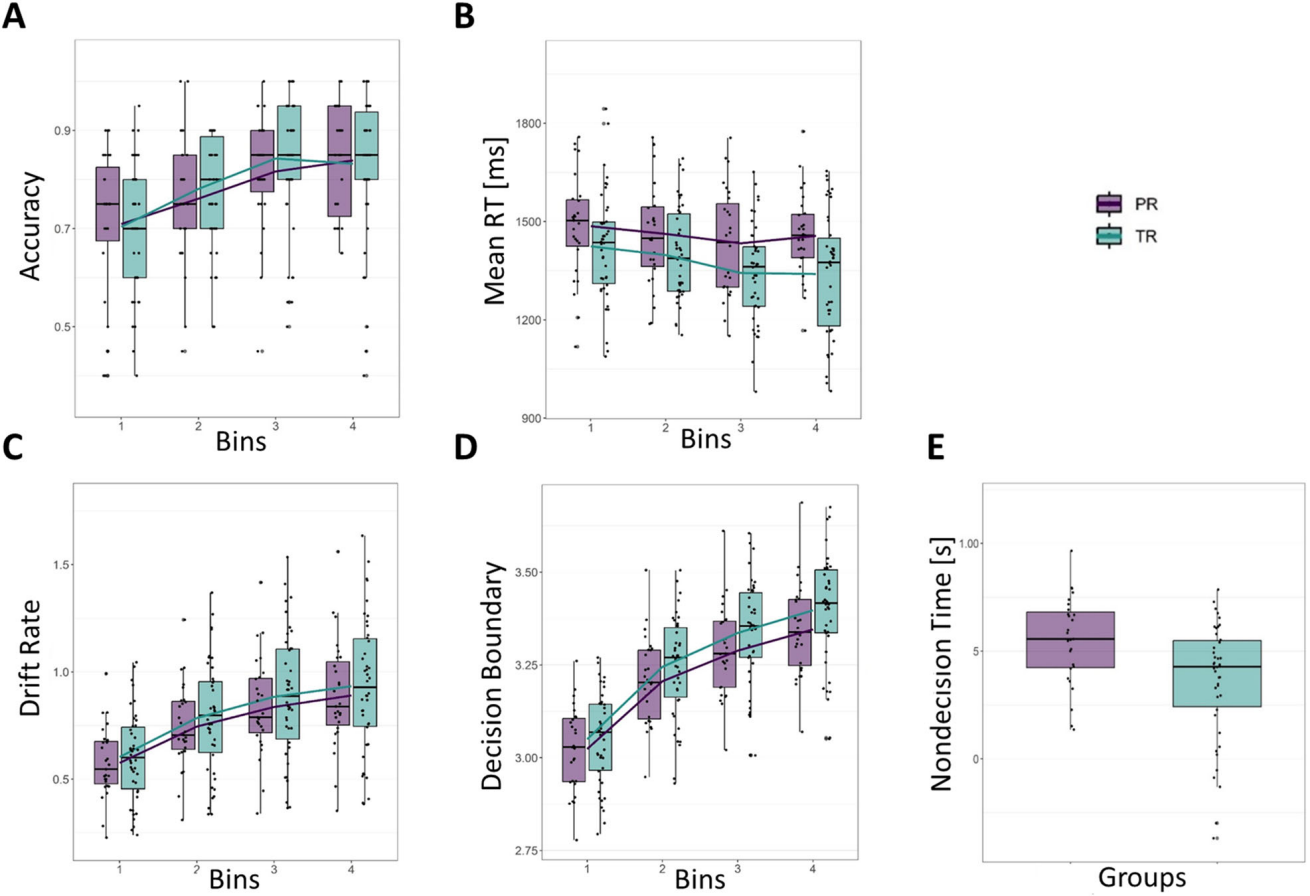
***A***, The overall increase in accuracy (proportion of hits, averaged across both runs) with learning is illustrated for both groups across bins. ***B***, RTs decreased with learning for both groups, as shown by the decline in averaged RT across bins. Divergent RT decrease with learning per bin indicating a slower decrease in PR than TR across learning. ***C***, Divergent drift rate increase with learning per bin indicating a lower increase in PR than TR over the LSS task. ***D***, Divergent decision boundary increase per bin indicating lower increase in PR than TR across learning. ***E***, Decreased nondecision time for TR as compared with PR. In purple children with poor (PR), in turquoise children with typical reading skills (TR). Additional LMMs comparing intermediate to strong readers (ISR: > 25th percentile range) with poor readers (reduced sample: *n* = 69), as well as an analysis of the full group (*n* = 80) using reading fluency as a covariate are presented in Extended Data Table 3-1 (for more information, please refer to the supplementary material available in the public repository at https://osf.io/g8rjx/). Significant positive correlations between reading fluency and RT and reading fluency and nondecision time are shown in Extended Data Figures 3-1 and 3-2.

The LMM analysis including the factors runs, bins, and groups revealed significant main effects of bin (*t*_(472)_ = 8.64, *p* < 0.001) on accuracy, indicating an increase in accuracy over time (Fig. 3*A*). No significant main effects of group (*t*_(239)_ = −0.01, *p* = 0.992) or interactions (*t*_(472)_ = −0.02, *p* = 0.982) with these factors were found for accuracy. Post hoc comparisons indicated that the mean scores for bin1 (*M* = 0.74, SE = 0.02), bin2 (*M* = 0.82, SE = 0.02), and bin3 (*M* = 0.86, SE = 0.02) differed significantly (all *p* < 0.027); however, bin3 and bin4 (*M* = 0.88, SE = 0.02) did not differ (*p* = 0.90).

10.1523/JNEUROSCI.1119-24.2025.f3-1 Figure 3-1 Positive correlation between response time for hits and reading fluency (mean of pseudoword and word reading percentiles SLRT-II). Since we hypothesised shorter RT with increasing reading skills, we conducted one-sided correlation analyses between RT and reading fluency (SLRT-II score; mean Word and Pseudoword reading percentile). Reading fluency skills correlated significantly negatively (*r* = - 0.362, *p* < 0.001) with RT for hits of FF-SS. Accuracy (number of hits) during both runs showed no significant correlation with reading-related tests. Download Figure 3-1, TIF file.

10.1523/JNEUROSCI.1119-24.2025.f3-2 Figure 3-2 Non-decision time correlated with reading fluency (mean of pseudoword and word reading percentiles SLRT-II). (SLRT-II, *r* = -0.344, *p* < 0.001) (Figure 1-2). Download Figure 3-2, TIF file.

10.1523/JNEUROSCI.1119-24.2025.t3-1 Table 3-1 **Additional LMMs including intermediate to strong (ISR) vs PR readers or the full sample and reading a continuous covariate of interest**. The results obtained after excluding 11 children whose reading scores fell within the 16th to 25th percentile, and those using the full sample with reading fluency as a covariate, were comparable to the findings reported for the core groups in the main text. For completeness, we provide all results in detail: please refer to the supplementary material available in the public repository at https://osf.io/g8rjx/. Download Table 3-1, XLSX file.

10.1523/JNEUROSCI.1119-24.2025.t3-2 Table 3-2 Association of drift rate and decision boundary with bin and group based on Bayesian statistics, in which the parameters were obtained by applying the RLDDM for all subjects. Download Table 3-2, XLSX file.

10.1523/JNEUROSCI.1119-24.2025.t3-3 Table 3-3 Modeling parameters obtained by RLDDMs including children with poor and typical reading skills separately (as well as t- and p-values of the comparison between groups) and the whole sample. Download Table 3-3, XLSX file.

10.1523/JNEUROSCI.1119-24.2025.t3-4 Table 3-4 Association of drift rate and decision boundary with bin and group based on frequentist statistics, in which the parameters were obtained by applying the RLDDM separately for each group. Download Table 3-4, XLSX file.

The LMM analysis for RT showed a significant main effect of bin (*t*_(472)_ = −5.68, *p* < 0.001) but not of group (*t*_(184.6)_ = 0.24, *p* = 0.815) on RT (Fig. 3*B*), indicating that children responded faster over time. Additionally, there was a significant group-by-bin interaction (*t*_(472)_ = 2.39, *p* = 0.017), indicating that children with poor reading skills showed a lower decrease as learning progressed compared with children with typical reading skills. When examining the post hoc contrasts with bin treated as an ordinal variable, the interaction effect appears to diminish, as none of the pairwise comparisons reached statistical significance (all *p* > 0.05). This discrepancy could be attributed to the increased sensitivity of the linear mixed model to detect interaction effects, even when they are subtle. While the interaction effect is present, its practical significance may be considered marginal, as evidenced by the small effect sizes in the post hoc contrasts. RT was also negatively correlated with reading fluency score (*r* = −0.362, *p* < 0.001; Extended Data Fig. 3-1).

#### RLDDM parameters

The LMM for drift rate (*v̂*), based on frequentist statistics, during bins showed a significant main effect of bin (*t*_(4792)_ = 100.83, *p* < 0.001) and of the group-by-bin interaction (bin × group: *t*_(4792)_ = −3.67, *p* < 0.001; Fig. 3*C*) indicating a lower increase in the drift rate across learning progression in children with poor reading skills. A Bayesian LMM for the association of drift rate with bin and group led to the same conclusions (Extended Data Table 3-2). Post hoc bin-wise comparisons showed that the effect was significant for bin differences: bin 1 versus 2 (*p* = 0.015), 1 versus 3 (*p* < 0.001), 1 versus 4 (*p* < 0.001), and 2 versus 4 (*p* = 0.015), with marginal means for poor readers for bin1, *M* = 0.76; bin2, *M* = 1.01; bin3, *M* = 1.15, and bin4, *M* = 1.22 (all SE = 0.06) and for typical readers for bin1, *M* = 0.82; bin2, *M* = 1.09, bin3, *M* = 1.24, and bin4, *M* = 1.31 (all SE = 0.05).

The decision boundary modification parameter (*ɑ*_mod_) indicated that, as trials progressed, the decision boundary was adjusted logarithmically in both groups (0 < *ɑ*_mod_ < 1). Interestingly, *ɑ*_mod_ was significantly lower in poor readers (*t*_(66.3)_ = −2.28, *p* = 0.026), indicating a flatter shape of the decision boundary adjustment across trials (Fig. 3*D*). This means that, compared with typical readers, poor readers showed a reduced increase of their decision boundary across time. Accordingly, this was also evident in an LMM for decision boundary (*a*_t_), based on frequentist statistics, with a significant main effect of bin (*t*_(4859)_ = 107.08; *p* < 0.001) and a significant group-by-bin interaction (*t*_(4859]_ = −3.82, *p* < 0.001); the main effect of group was nonsignificant (*t*_(78.91) _= −0.62, *p* = 0.534). A Bayesian LMM for the association of decision boundary with bin and group led to the same conclusions (Extended Data Table 3-2). Post hoc bin-wise comparisons showed that the interaction was particularly present in bin 1 versus 2 (*p* = 0.023), 1 versus 3 (*p* < 0.001), and 1 versus 4 (*p* < 0.001) as well as 2 versus 4 (*p* = 0.022), with marginal means for poor readers for bin1, *M* = 3.02; bin2, *M* = 3.21; bin3, *M* = 3.29, and bin4, *M* = 3.35 (all SE = 0.03) and for typical readers for bin1, *M* = 3.05; bin2, *M* = 3.24; bin3, *M* = 3.33, and bin4, *M* = 3.40 (all SE = 0.02).

We conducted an additional analysis to examine the association of drift rate and decision boundary with bin and group, using parameters obtained through RLDDMs performed separately for each group, consistent with the approach of Manning et al. (2022). The descriptive statistics are presented in Extended Data Table 3-3, and the results of the association of drift rate and decision boundary with bin and group are shown in Extended Data Table 3-4. While the significant effects of bin and the interaction between bin and group for both drift rate and decision boundary align closely with those obtained from a single RLDDM applied to the entire sample, a new finding emerged: a significant group effect for the decision boundary. Specifically, the decision boundary was lower for children with poor reading skills compared with those with typical reading skills when using parameters derived from group-specific RLDDMs. We attribute this result to the increased sensitivity to the group-specific approach, as performing RLDDM separately for each group yielded stronger *t* values compared with analyses based on parameters from a single RLDDM.

We further compared RLDDM parameters between children with poor and typical reading skills. Using frequentist statistics based on subject-specific mean values, we found that children with poor reading skills exhibited a significantly higher nondecision time (*τ*) compared with those with typical reading skills (*t*_(67)_ = 3.049, *p* = 0.003; Fig. 3*E*). However, no significant group differences were observed for other parameters, namely, learning rate (*η*), decision boundary (*ɑ*), drift rate (*v̂*), and starting point (*z*; all *ps* > 0.1). To account for trial-wise variance present in certain parameters (i.e., *τ* and *α*), we also compared RLDDM parameters using Bayesian statistics. This analysis corroborated the results of frequentist statistics, showing higher nondecision time (*τ*) in children with poor reading skills compared with those with typical reading skills [posterior probability (PP) = 1]. No significant group differences were found for learning rate (*η*; PP = 0.59), drift rate (*v̂*; PP = 0.82), or starting point (*z*; PP = 0.59). Evidence for group differences in decision boundary (*α*) was deemed moderate but not strong (PP = 0.91). Additionally, nondecision time (*τ*) showed a negative correlation with reading fluency scores (*r* = −0.344, *p* = 0.002; Extended Data Fig. 3-2).

Of note, all results of the present LMM analyses were also replicated with the sample excluding the 11 children with reading skills in the 16th–25th percentile range as well as with the reading score as continuous covariate (refer to the supplementary material available in the public repository at https://osf.io/g8rjx/; for an overview, see Extended Data Table 3-1).

### Changes in activation and connectivity during learning

#### Group differences in feedback processing between children with poor and typical reading skills

**Figure 4.**
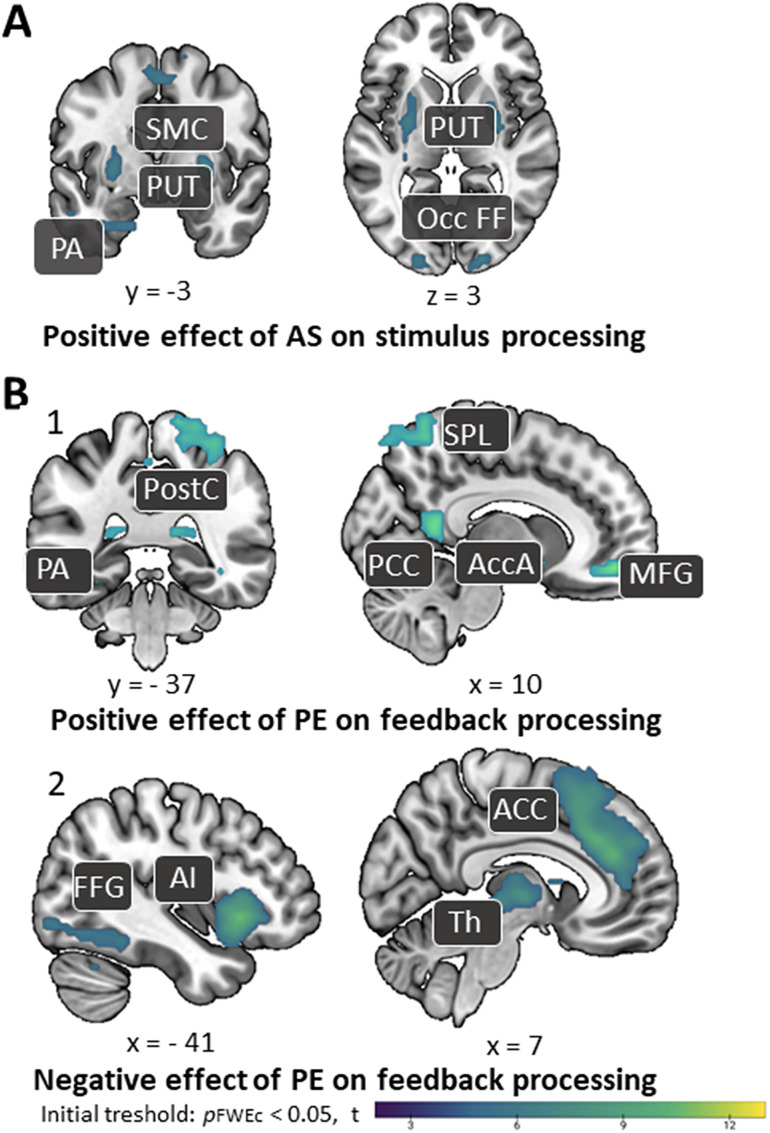
A model-based analyses: positive effect of ***A*S** on stimulus processing; supplementary motor cortex, putamen, precuneus, parahippocampal area, occipital fusiform form area. ***B1***, Positive effect of **PE** on feedback processing; angular gyrus, parahippocampal area, superior parietal lobe, posterior cingulate cortex, nucleus accumbens, middle frontal gyrus, postcentral gyrus; ***B2***, Negative effect of **PE** on feedback processing; fusiform gyrus, anterior insula, thalamus, anterior cingulate cortex, middle temporal gyrus. Initial threshold *p*FWE = 0.05, *p*FWEc < 0.05. Abbreviations: PUT, bilateral putamen; Thp, thalamus proper; Pal, pallidum; STG, superior temporal gyrus; AI, anterior insula; MFG, bilateral middle frontal gyrus; Occ, calcarine cortex/bilateral occipital poles/inferior occipital/lingual; Str, bilateral striata (putamen, caudate); PA, parahippocampal area; Occ FF, occipital fusiform form area; PCC, posterior cingulate cortex; AccA, nucleus accumbens area; PostC, postcentral gyrus; FFG, fusiform gyrus; STS, superior temporal sulcus; PAC, primary auditory cortex; ACC, anterior cingulate cortex; SPL, superior parietal lobe; SMC, supplementary motor cortex; Th, thalamus. Brain images of the conventional analyses (stimulus-baseline, feedback-baseline, and group comparison) are shown in Extended Data Figures 4-1 and 4-2. Tables presenting the *t*-maps from both the conventional and model-based analyses can be found in Extended Data Tables 4-1, 4-2, 4-4, and 4-5. VWFA activity during stimulus processing in children with typical and poor reading skills are displayed in Extended Data Figures 4-4 and 4-5. The literature-based VWFA ROI is illustrated in Extended Data Figure 4-3, with the corresponding references and coordinates provided in Extended Data Table 4-3.

Whole-brain analyses on overall activation related to stimulus and feedback processing in the whole group are summarized in the supplement (Extended Data Table 4-1, Fig. 4-1). Group comparisons between children with typical or poor reading skills yielded no differences. However, when we excluded the intermediate readers, we found a significant difference during feedback processing in the right angular gyrus (AnG), the precentral gyrus (PreC), and the superior frontal gyrus (SFG; Extended Data Fig. 4-2, Table 4-2) but none during stimulus processing.

#### Association between fMRI activation and learning parameters

The parametric modulations of AS and PE at the time of stimulus and feedback presentation yielded the following results: We observed a positive modulation of AS on activation in bilateral superior frontal gyri and precentral gyri, right inferior frontal gyrus, and postcentral gyrus, left parahippocampal and occipital fusiform gyri, and bilateral putamen (Fig. 4*A*, Extended Data Table 4-4) during FF–SS learning. Furthermore, for feedback processing trials, a positive effect of PE on activation in the angular gyrus, parahippocampal gyri, posterior cingulate cortex, middle frontal gyrus, nucleus accumbens (part of basal ganglia), postcentral gyrus, and superior parietal lobe was observed (Fig. 4*B*1, Extended Data Table 4-5). Finally, a negative effect of PE on activation in bilateral anterior insulae, thalami, middle temporal gyrus, fusiform gyri, and inferior occipital gyri, as well as the right inferior temporal gyrus and middle frontal gyrus, anterior cingulate cortex, supplementary motor area, and precentral gyrus was found (Fig. 4*B*2, Extended Data Table 4-5).

10.1523/JNEUROSCI.1119-24.2025.f4-1 Figure 4-1 **Conventional analyses; Left**: brain activation at stimulus presentation: bilateral transverse temporal gyri, bilateral precentral gyri, bilateral putamen, thalamus, pallidum, superior frontal gyrus, superior temporal gyrus, anterior insula, bilateral middle frontal gyri, calcarine cortex, inferior and superior occipital. **Right:** brain activation during feedback processing: bilateral angular gyri, bilateral striatum (putamen, caudate), bilateral anterior insulae, bilateral middle frontal gyri, bilateral middle temporal gyri, bilateral superior frontal gyri, bilateral occipital poles/inferior occipital/lingual gyrus. The whole brain analyses of stimulus and feedback processing in the whole group of children (n = 80) indicated activation of an extended network processing the sensory information such as the bilateral auditory and visual regions, further in the anterior insulae (AI), putamen (PU), and superior parietal cortex, pre- and postcentral regions (PreC/PostC), anterior cingulate cortex (ACC) and parts of the right striatum (Str), during audio-visual stimulation. During feedback processing the bilateral angular gyri (AnG), striatal regions including the PU, AI, middle frontal gyri (MFG), and occipital poles were activated (Occ) (see Table 4-1). Download Figure 4-1, TIF file.

10.1523/JNEUROSCI.1119-24.2025.f4-2 Figure 4-2 **Group comparison of children with intermediate to strong (ISR, n** **=** **42) vs poor reading (PR, n** **=** **27) skills:** feedback processing vs baseline; significant activation in right Precuneus, right Angular Gyrus, and right Superior Frontal Gyrus. Cluster defining threshold *p_uncorr._* = 0.001, cluster correction *pFWEc* < 0.05. **Abbreviations:** SFG = Superior Frontal Gyrus, AnG = Bilateral Angular Gyrus, PreC = Precentral Cortex. Additional group comparisons between children with intermediate to strong (n = 42) or poor reading skills (n = 27) yielded differences during feedback processing in the right angular gyrus (AnG), the precentral gyrus (PreC), and the superior frontal gyrus (SFG) (Table 4-2) but none during stimulus processing. Download Figure 4-2, TIF file.

10.1523/JNEUROSCI.1119-24.2025.f4-3 Figure 4-3 **Literature-based mask of the Visual Word Form Area.** Additional region of interest (ROI) analysis of the VWFA was performed to examine whether children with typical and with poor reading skills show differences in the visual processing of the false font characters during the LSS task. A literature-based VWFA mask was used (c.f. (Haugg et al. 2023)) which was created by defining spheres with different radii around the activation peaks reported in several articles on VWFA listed below using the MarsBaR toolbox for SPM (MARSBAR V0.41, http://marsbar.sourceforge.net/). These spherical ROIs were then combined to form a joint VWFA mask (see Table 4-3). Download Figure 4-3, TIF file.

10.1523/JNEUROSCI.1119-24.2025.f4-4 Figure 4-4 **Group differences on a trend level between typical and poor readers**. From the joint VWFA ROI, we extracted beta values using MarsBaR and conducted a two-sample t-test to compare activation in children with typical versus poor reading skills. There was no significant difference between groups in the activation of the VWFA during the LSS task. However, a statistical trend indicated that children with typical reading skills had marginally higher BOLD signal in the VWFA than children with poor reading skills (Figure 1-7). This was similar for the core sample of 80 children (*t*(78) = -1.709, *p* = 0.091) as well as after excluding the 11 children whose reading skills fell within the 16th to 25th percentile (*t*(67) = -0.670, *p* = 0.051). Download Figure 4-4, TIF file.

**Figure 5.**
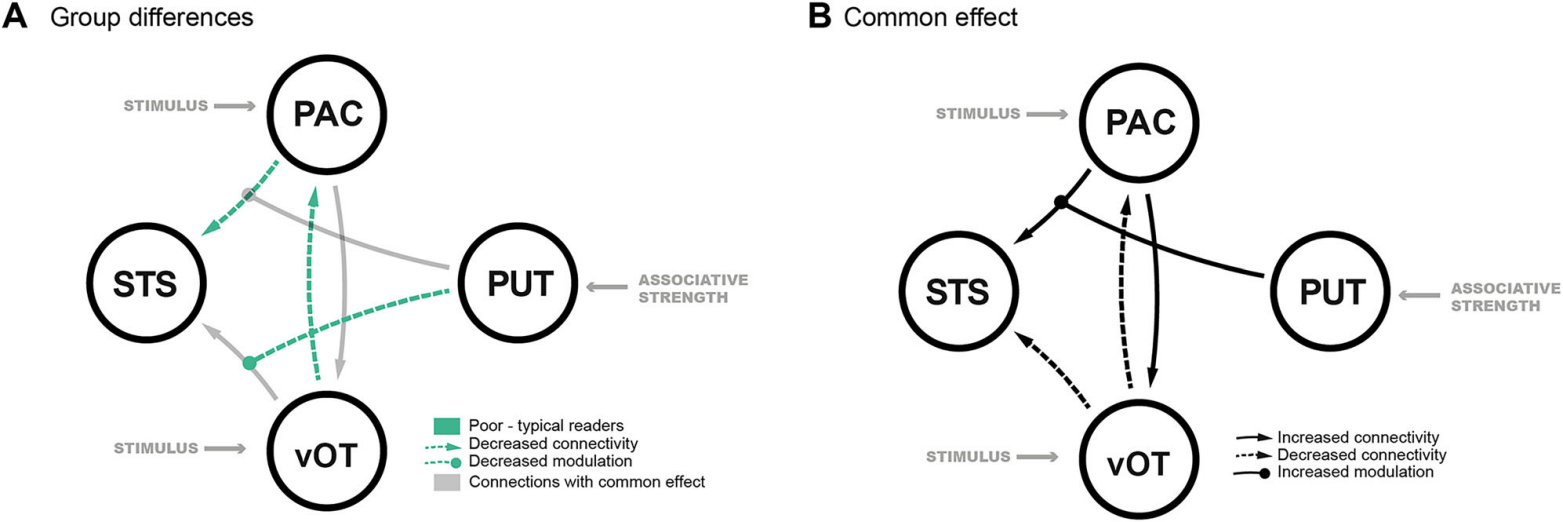
***A***, Group difference in connectivity between children with typical and with poor reading skills. Gray lines represent baseline connectivity patterns observed across all children, while green lines indicate significant deviations in connectivity specific to the PR group. The decreased connectivity and modulations are shown as dashed lines. ***B***, Common effects in effective connectivity across the whole group, regardless of reading ability. Solid lines represent positive connectivity and modulation, and dashed lines show inhibitory connectivity. Group differences in connectivity between children with intermediate and poor reading skills are illustrated in Extended Data Figure 5-1, while Extended Data Figure 5-2 shows connectivity across all children, using reading score as a continuous variable. The corresponding *t*-maps are provided in Extended Data Tables 5-1 and 5-2.

10.1523/JNEUROSCI.1119-24.2025.f4-5 Figure 4-5 **VWFA activation in children with typical and with poor reading skills.** VWFA ROI in yellow, TR = children with typical reading skills (n = 53), PR = children with poor reading skills (n = 27). Download Figure 4-5, TIF file.

10.1523/JNEUROSCI.1119-24.2025.t4-1 Table 4-1 **Results of conventional fMRI analysis: stimulus vs baseline and feedback vs baseline (n** **=** **80).** Significant clusters on the whole-brain level in the contrast stimulus vs. baseline. This analysis aimed at revealing the task-relevant network and guided the selection of ROIs for further analysis (DCM). Significance level at cluster defining initial threshold *pFWE* = 0.05, cluster correction *pFWEc* < 0.05, all clusters k > 5 are tabulated, G = gyrus, L = left; R = right, m = medial, MC = motor cortex. The extended clusters in the bilateral transverse temporal gyri included the following brain structures: planum temporale, parietal and frontal opercula, posterior and anterior insulae, middle frontal gyri, precentral gyri, putamen, supplementary motor cortices, superior temporal gyri. G = Gyrus, WM = White Matter. Download Table 4-1, XLSX file.

10.1523/JNEUROSCI.1119-24.2025.t4-2 Table 4-2 **Two-sample t-test between ISR and PR.** Group comparison intermediate to strong (ISR) vs poor for contrast feedback vs baseline. Cluster defining threshold *p_uncorr._* = 0.001, cluster correction *pFWEc* < 0.05, k = 69. G = Gyrus, WM = White Matter, ISR n = 42, PR, n = 27. Download Table 4-2, XLSX file.

10.1523/JNEUROSCI.1119-24.2025.t4-3 Table 4-3 **Literature based VWFA ROI:** Coordinates and radii of the spheres chosen based on literature. Additional region of interest (ROI) analysis of the VWFA was performed to examine whether children with typical and poor reading skills show differences in the visual processing of the false font characters during the LSS task. A literature-based VWFA mask was used (c.f. (Haugg et al. 2023)) which was created by defining spheres with different radii around the activation peaks reported in several articles on VWFA listed below using the MarsBaR toolbox for SPM (MARSBAR V0.41, http://marsbar.sourceforge.net/). These spherical ROIs were then combined to form a joint VWFA mask (see Table 4-3). Download Table 4-3, XLSX file.

10.1523/JNEUROSCI.1119-24.2025.t4-4 Table 4-4 **Results of model-based fMRI analysis (n** **=** **80): Positive and negative parametric modulation of AS on stimuli.** Significant clusters for the parametric modulation of positive (pos) and negative (neg) associative strength on stimulus processing. Cluster defined initial threshold *pFWE* = 0.05, cluster corrected *pFWEc* < 0.05, clusters exceeding k > 5 are listed, G = gyrus, L = left; R = right, MC = motor cortex, WM = white matter. Download Table 4-4, XLSX file.

10.1523/JNEUROSCI.1119-24.2025.t4-5 Table 4-5 **Results of model-based fMRI analysis: Parametric modulation of positive and negative PE on feedback (n** **=** **80).** Significant clusters for in the parametric modulation of positive (pos) and negative (neg) Prediction error (PE) on feedback processing. Cluster defined initial threshold *pFWE* = 0.05, cluster corrected *pFWEc* < 0.05, clusters exceeding k > 3 are listed, G = gyrus, L = left; R = right, MC = motor cortex, WM = white matter, G = Gyrus. Download Table 4-5, XLSX file.

While the data did not reveal brain regions with a negative effect of AS on stimulus processing, we also found no significant group differences in how AS or PE modulated the BOLD signal.

#### Altered connectivity during learning in children with poor reading skills using dynamic causal modeling

Using DCM, we found diminished striatal modulation of audiovisual networks in children with poor reading skills during FF–SS learning (Fig. 5*A*, Table 4) as detailed below.

10.1523/JNEUROSCI.1119-24.2025.f5-1 Figure 5-1 **A Group difference in connectivity between children with intermediate to strong (ISR) and with poor reading skills.** Gray lines represent baseline connectivity patterns observed across all children, while green lines indicate significant deviations in connectivity specific to the PR group. Decreased connectivity and modulations are shown as dashed lines. **B.** Common effects in effective connectivity across the whole group, regardless of reading skills. Solid lines represent positive connectivity and modulation, dashed lines show inhibitory connectivity. Download Figure 5-1, TIF file.

10.1523/JNEUROSCI.1119-24.2025.f5-2 Figure 5-2 **DCM with reading sore as a continuous variable. A.** Effect of reading skills in the whole group**. B.** Common effects in the whole group (n = 75). We replicated our DCM analyses with the whole (n = 75) group of children and with reading score as a continuous variable. Overall, similar common effects were found when repeating the analysis with the reading fluency scores (instead of group assignments) as a predictor. We found a significant effect of reading ability on the vOT’s afferent connections and its self-connection (i.e. the input sensitivity of the region). Lower reading fluency scores were related to decreased self-inhibition of the vOT and connectivity from STS to vOT, and increased connectivity between PAC and vOT (Figure 1-10). In this model, the effect of the reading fluency score on the striatal modulation did not reach statistical significance (Table 5-2). Download Figure 5-2, TIF file.

10.1523/JNEUROSCI.1119-24.2025.t5-1 Table 5-1 **Connectivity parameters obtained by Bayesian model averaging**. Between-region connections are in units of Hz. Self-inhibition parameters, where the source and target are the same, are the log of scaling parameters that multiply up or down the default value −0.5  Hz. Posterior probabilities are given in the brackets. PR, N = 27; TR, N = 38. AS = association strength; PAC = primary auditory cortex; PUT = putamen; STS = superior temporal sulcus; vOT = ventral occipito-temporal cortex. ^a^ only means for all participants to be tested. Download Table 5-1, XLSX file.

10.1523/JNEUROSCI.1119-24.2025.t5-2 Table 5-2 **Connectivity parameters obtained by Bayesian model averaging using standardised SLRT-II (mean of word and pseudoword reading percentiles) as a covariate**. The posterior probability is reported in brackets. Between-region connections are in units of Hz. Self-inhibition parameters, where the source and target are the same, are the log of scaling parameters that multiply up or down the default value −0.5  Hz. Posterior probabilities are given in the brackets. *n* = 75. PAC, primary auditory cortex; Association strength = AS; PUT = putamen; SLRT-II, Salzburger Lese-/Rechtschreibtest; STS = superior temporal sulcus; vOT, ventral occipito-temporal cortex. *Reading fluency score of SLRT-II word and pseudoword reading. Download Table 5-2, XLSX file.

**Table 4.** Connectivity parameters obtained by Bayesian model averaging

Connection type	Common	Group	Age	Handed	Sex
**Endogenous parameters**					
vOT-PAC	−0.281 (1)	−0.083 (1)	-	-	−0.152 (1)
PAC-vOT	0.239 (1)	0.035 (0.55)	-	0.179 (1)	-
STS-PAC	-	0.034 (0.60)	-	-	-
PAC-STS	0.461 (1)	−0.082 (1)	0.043 (0.54)	0.174 (1)	0.059 (0.58)
vOT-STS	−0.225 (1)	-	-	0.107 (1)	
STS-vOT	-	−0.030 (0.56)	-	-	−0.134 (1)
**Self-inhibition parameters**					
PUT-PUT	-	-	-	-	0.073 (0.67)
vOT-vOT	−0.351 (1)	-	−0.098 (1)	−0.045 (0.53)	-
PAC-PAC	-	-	-	−0.146 (1)	−0.270 (1)
STS-STS	0.049 (0.56)	-	0.126 (1)	0.224 (1)	0.209 (1)
**Modulatory parameters from PUT**					
vOT-PAC	-	−0.526 (0.71)	0.368 (0.51)	−1.155 (0.97)	0.428 (0.50)
PAC-vOT	-	-	-	-	1.193 (1)
STS-PAC	-	−0.542 (0.72)	-	-	-
PAC-STS	0.871 (0.99)	-	-	-	-
vOT-STS	-	−1.407 (1)	−0.624 (0.74)	-	-
STS-vOT	-	-	-	-	-
**Input parameters** ^a^					
Stimulus presentation-vOT	0.149 (0.94)	-	-	-	-
Stimulus presentation-PAC	1.117 (1)	-	-	-	-
AS-Putamen	1.288 (1)	-	-	-	-

Between-region connections are in units of Hz. Self-inhibition parameters, where the source and target are the same, are the log of scaling parameters that multiply up or down the default value −0.5 Hz. Posterior probabilities are given in the brackets. PR, *N* = 27; TR, *N* = 48. AS, association strength; PAC, primary auditory cortex; PUT, putamen; STS, superior temporal sulcus; vOT, ventral occipitotemporal cortex.

a Only means for all participants to be tested.

We observed a significant common effect across children with both poor and typical reading skills on the intrinsic connections (*A*-matrix). Specifically, we identified significant bidirectional connections between the primary auditory cortex (PAC) and the ventral occipitotemporal cortex (vOT), as well as connections from both the vOT and PAC to the superior temporal sulcus (STS; Fig. 5*B*, Table 4). Additionally, we detected significant striatal modulation during stimulus presentation on the connection from the vOT to the STS. The maximum a posteriori first-level DCM estimates for this connection were *M* = 0.10 for children with typical reading skills and *M* = −0.13 for children with poor reading skills. Importantly, children with poor reading skills exhibited significantly decreased striatal modulation of connectivity between the primary auditory cortex (PAC) and the ventral occipitotemporal cortex (vOT; Fig. 5*A*, Table 4). Additionally, these children showed weaker efferent connections from the vOT to PAC and from PAC to the superior temporal sulcus (STS).

Further, DCM analyses using the sample excluding 11 children with reading skills in the 16th–25th percentile range and using the whole sample with a covariate of reading fluency, summarized in the extended data (Extended Data Tables 5-1, 5-2, Figs. 5-1, 5-2), largely corresponded to the results of the full sample (Table 4, Fig. 5).

## Discussion

This study uses computational modeling to examine neural changes linked to LSS learning, highlighting its role in developing brain networks for fluent reading in children with varying reading abilities. The core results of our study are twofold: Firstly, we show that while both children with typical (TR) and poor reading (PR) skills can quickly learn artificial LSS correspondences, PR have longer nondecision times and slower increases in drift rate and decision boundary across learning, indicating a different learning trajectory. Secondly, model-based fMRI analyses showed alterations in effective connectivity among the ventral occipitotemporal cortex (vOT), auditory cortex (PAC), and superior temporal sulcus (STS), along with diminished striatal modulation of the vOT-STS connection in PR during the learning task. These findings emphasize the role of interactions within specialized neural networks in forming audiovisual LSS representations and reveal alterations in the learning trajectory and network connectivity in PR, suggesting a potential mechanism underlying children's difficulties with subsequent integration and/or automation (Hahn et al., 2014; Fraga González et al., 2016) and/or application of novel LSS representations (Vaessen et al., 2009; Blomert, 2011; Aravena et al., 2013) during reading.

Overall, children quickly learned false font–speech sound associations, becoming more accurate and faster with their decisions over time, regardless of their reading skills, consistent with previous studies (Aravena et al., 2013; Law et al., 2018). Behavioral model analyses provided deeper insights, revealing that PR exhibited altered learning patterns, in line with earlier findings (Snowling, 1980; Blau et al., 2009; Blomert, 2011; Aravena et al., 2018; Karipidis et al., 2018). Specifically, these children showed lower increases in drift rate and decision boundary compared with TR. The findings of prolonged response times and slower increases in drift rate indicate that PR take longer to accumulate evidence to establish letter–sound mappings. This may result from slower cognitive processing speed or inefficiencies in integrating information. The different rates of change in the decision boundary across learning trials between groups suggest that TR seem to need comparably less evidence to make a decision. As they learn, TR become increasingly confident in processing letter–sound mappings, requiring less evidence to make accurate choices. In contrast, PR may take longer to adapt their decision-making process, adopting a more conservative approach that requires additional trials to build confidence. This suggests that PR may develop noisier and less robust LSS mappings during learning, necessitating more evidence for effective decision-making. Further, attentional challenges (Gilger et al., 1992; Laasonen et al., 2009; Boada et al., 2012), where PR struggle to maintain focus, may contribute to slower decision-making. The increased uncertainty may also indicate a faster decay of implicit memory, resulting in less robust representations (Jaffe-Dax et al., 2018; Pleisch et al., 2019). Difficulties with working memory, particularly in retaining phonological information related to speech sounds (Pennington et al., 1990; Gathercole and Baddeley, 1993; De Carvalho et al., 2014; Alt et al., 2022), could hinder their ability to hold and manipulate the information necessary for effective decision-making during learning tasks.

Alterations in decision-making processes in children with dyslexia have been reported across various tasks, including low-level sensory motion processing and probabilistic decision-making (O'Brien and Yeatman, 2021; Stefanac et al., 2021; Manning et al., 2022; Pereira et al., 2022). O'Brien and Yeatman (2021) applied a drift-diffusion model to a visual motion processing task in a similarly aged group of children with varying reading abilities. While we found a difference in the change in drift rate with learning between groups, they found an association between drift rate and reading skills, specifically in children with high phonological awareness. Further, our findings of higher nondecision time and slower decision-making in PR might stem from differences in perceptual processing (Stefanac et al., 2021) or challenges in perceptual encoding and motor execution (Theisen et al., 2021). Differences in LSS association learning may also be influenced by auditory category learning deficits in children with dyslexia (Gabay et al., 2015; 2023; Gabay and Holt, 2015; Roark et al., 2024), particularly in noisy environments like MR scanners. Differences in task designs between studies however make direct comparisons challenging.

Taken together, our findings suggest that PR experience higher uncertainty due to the establishment of weaker or noisier links between newly learned letter–sound associations. Such deficient audiovisual representations could stem from impaired interactions between unisensory and audiovisual integration regions during learning. Using dynamic causal modeling, our effective connectivity analysis thus examined the functional coupling of core nodes in the audiovisual learning network (Li et al., 2019). Li and colleagues’ study on audiovisual integration in adults (Li et al., 2019) suggested distinct optimal connectivity models for processing informative and uninformative sounds, whereby informative sounds involved both the unisensory–multisensory and unisensory–unisensory pathways. Our results suggest that disruptions in these pathways (vOT-PAC, vOT-STS, and PAC-STS) may underlie impairments in forming robust LSS associations in PR. During LSS learning, we found evidence of striatal gating influencing the flow of information between auditory, visual, and audiovisual integration areas across the entire group of children. Across both groups, striatal modulation of the connection from the PAC to the STS integration region indicates that the current learning state of a LSS association is related to the synaptic strength between those regions and underlines the striatum's critical role during associative learning (den Ouden et al., 2009). Strikingly, in PR, the striatal gating between visual (vOT) and audiovisual integration areas was inhibitory as compared with TR showing excitatory modulation between these regions on the group level. Increases in associative strength and the linked development of more robust predictive representations may enhance salience and discrimination of the false font characters and facilitate their mapping onto the corresponding speech sounds. However, impaired striatal gating between vOT and integrative regions during learning may affect this process. This altered connectivity during audiovisual integration between visual and audiovisual regions might underlie the observed less efficient and thus slower decision-making process across learning, indicative of the reported audiovisual integration deficit and the subsequent problem in forming robust LSS mappings (Snowling, 1980; Blomert and Willems, 2010; Peterson and Pennington, 2012; Peterson and Pennington, 2015). PR additionally showed weaker connectivity from auditory to audiovisual regions and from visual to auditory processing regions. Learning LSS associations relies on dynamic coupling between these regions, enabling synchronization of visual with auditory signals in vOT and PAC and further integration in the STS. Consequently, reduced connectivity and altered information flow within this network, combined with striatal gating deficits, likely impair the binding of letters and speech sounds, disrupting the formation of coherent multisensory representations (Richlan et al., 2011; Richlan, 2014) or access to stored representations (Boets et al., 2013).

Finally, parametric modulations examined how changes in AS and PE during stimulus or feedback processing, respectively, impact on activation throughout learning. AS is updated by PE on each trial and thus reflects the quality of associations formed based on past experiences. Our data largely converge with recent findings on AS and PE processing during LSS learning in adults (Fraga Gonzàlez et al., 2025). AS modulated activation in a network associated with visual processing, learning, memory, motor coordination, and planning (Stark et al., 2018; Gale et al., 2021), including increased occipital activation suggesting visual specialization to characters (Fraga Gonzàlez et al., 2025). Positive effects of PE were observed in parietal, middle frontal, basal ganglia, and hippocampal regions, while negative effects of PE emerged in the anterior cingulate and insula, among other regions. These findings support the contribution of these regions to PE encoding and evaluation during learning and suggest that regions processing the corrective impact of feedback assess stimulus salience, potentially triggering adaptation in specialized print-processing regions like the left vOT (Knutson et al., 2001; Preuschoff et al., 2008; van Kememade et al., 2017; Fraga González et al., 2025).

As potential limitation, it is essential to consider that different factors may have influenced our behavioral and MRI results. To assess the potential impact of fatigue, we examined block repetitions, omission rates, and motion parameters during fMRI scanning. Analyses revealed no significant differences between TR and PR in terms of block repetitions and omissions during learning. Although motion was higher in PR and increased with task duration, both groups showed comparable changes, suggesting that increased fatigue, despite being a potentially more cognitively demanding task for PR, did not substantially affect our results. Further, despite evidence that difficulties in LSS learning in dyslexia are related to diminished neural activity in specific regions such as the left vOT (Blomert, 2011; Richlan, 2014; Žarić et al., 2014; Norton et al., 2015; Richlan, 2019), our study found no major activation differences related to reading skills, with only marginal variations of activation in left vOT (Extended Data Figs. 4-1, 4-4), which may indicate constrains to detect subtle group differences due to limited sample sizes.

In summary, although children generally demonstrate an adept association between false font characters and speech sounds, detailed behavioral analyses reveal deficiencies in evidence accumulation and confidence for decision-making, in PR, particularly as learning progresses. This was accompanied by reduced interactions among brain regions in the audiovisual learning network and altered modulatory effects of associative strength (AS) by the putamen on the feedforward connectivity between visual letter processing and multisensory integration areas, implying disrupted connectivity in forming new audiovisual letter representations. In conclusion, our findings suggest a potential mechanism whereby disrupted interactions within the brain network for audiovisual learning during the initial phases of reading development could impact the formation of robust LSS representations and their subsequent utilization during reading.
