# Supplementary figures and images for: Toward a Mechanistic Understanding of Reading Difficulties: Deviant Audiovisual Learning Dynamics and Network Connectivity in Children with Poor Reading Skills
Source: J Neurosci. 2025 Feb 27;45(17):e1119242025. doi: 10.1523/JNEUROSCI.1119-24.2025 (PMC12019146; doi:10.1523/JNEUROSCI.1119-24.2025)

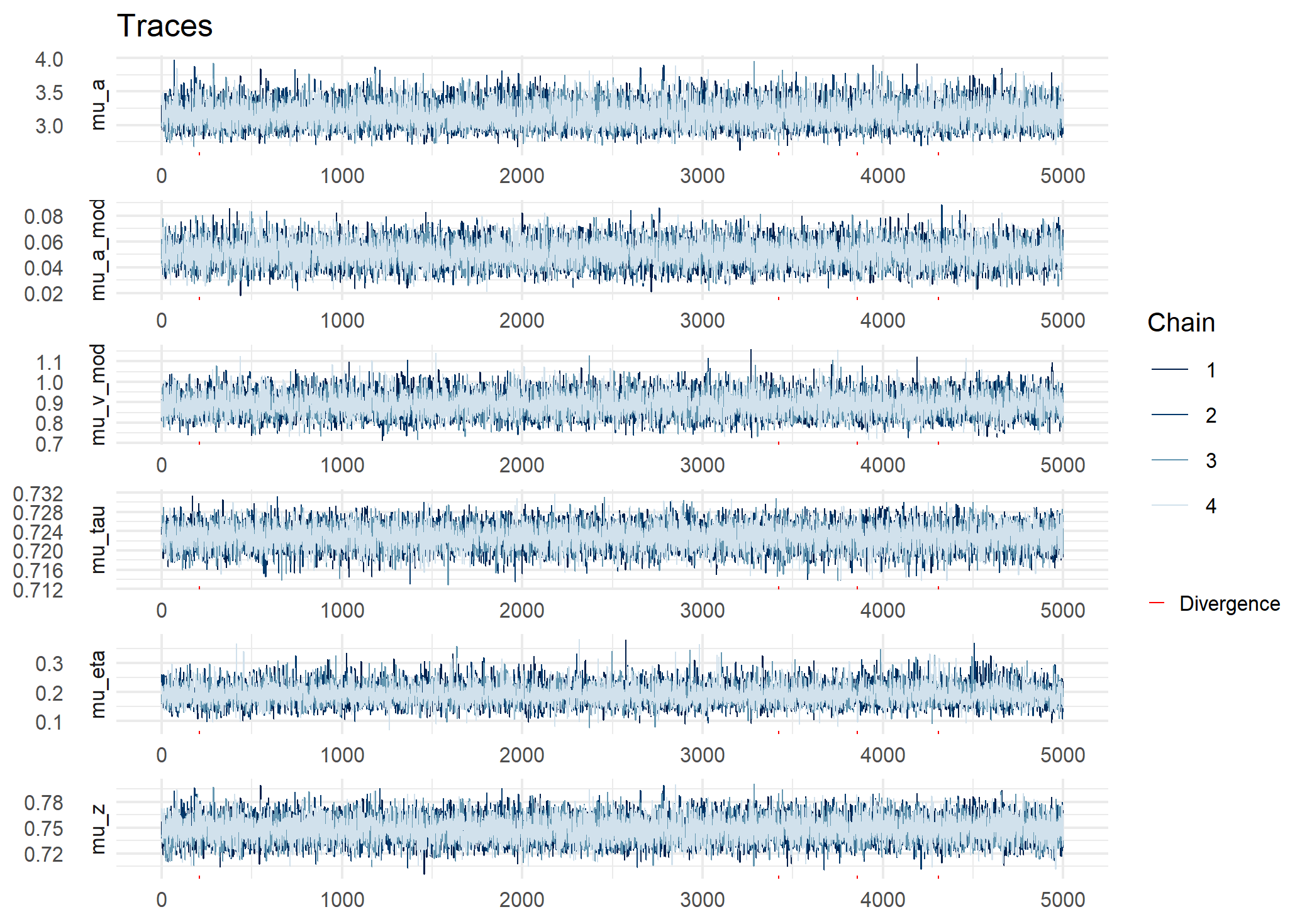

Supplement: Figure 2-1 — Traces of Markov chains for the group parameters. This figure displays the Markov chain Monte Carlo (MCMC) traces for key group parameters in our drift diffusion model analysis. Each panel represents a different parameter, showing how the estimates evolve over the course of the MCMC sampling process. Each trace represents 5’000 retained post-warmup samples from four independent chains. Download Figure 2-1, TIF file. [file jneuro-45-e1119242025-s010.tif]

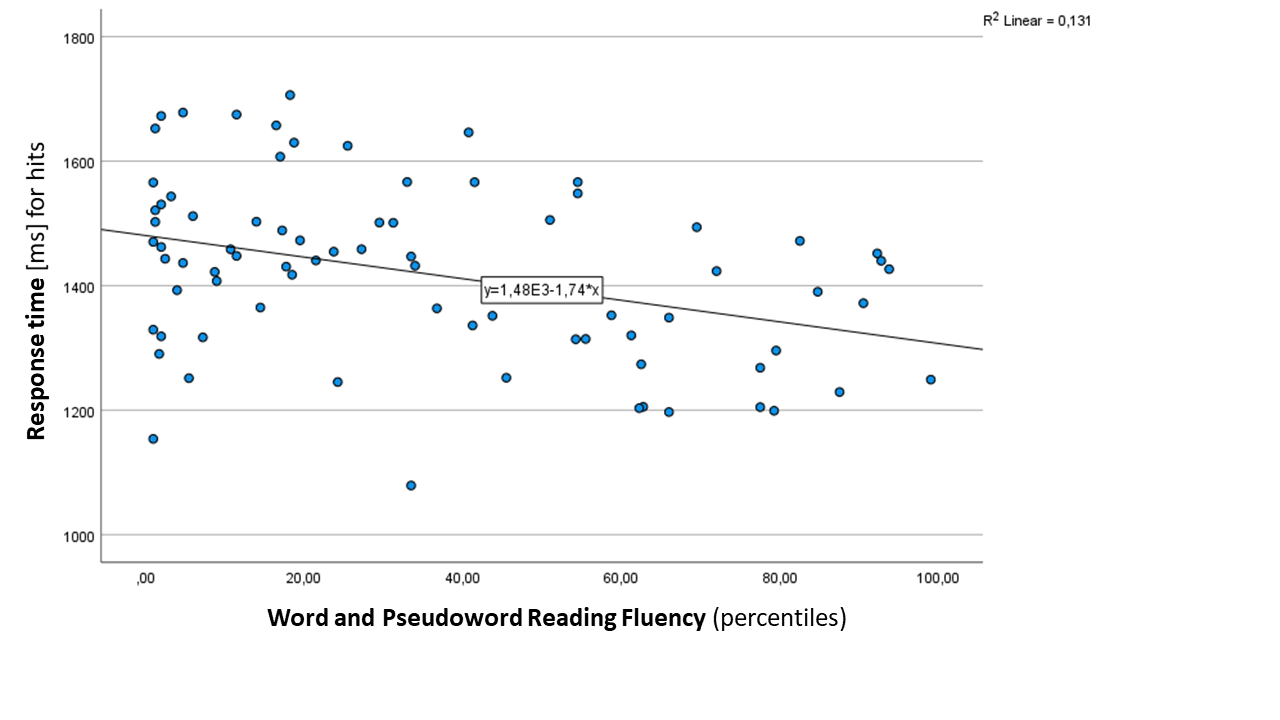

Supplement: Figure 3-1 — Positive correlation between response time for hits and reading fluency (mean of pseudoword and word reading percentiles SLRT-II). Since we hypothesised shorter RT with increasing reading skills, we conducted one-sided correlation analyses between RT and reading fluency (SLRT-II score; mean Word and Pseudoword reading percentile). Reading fluency skills correlated significantly negatively (r = - 0.362, p < 0.001) with RT for hits of FF-SS. Accuracy (number of hits) during both runs showed no significant correlation with reading-related tests. Download Figure 3-1, TIF file. [file jneuro-45-e1119242025-s011.tif]

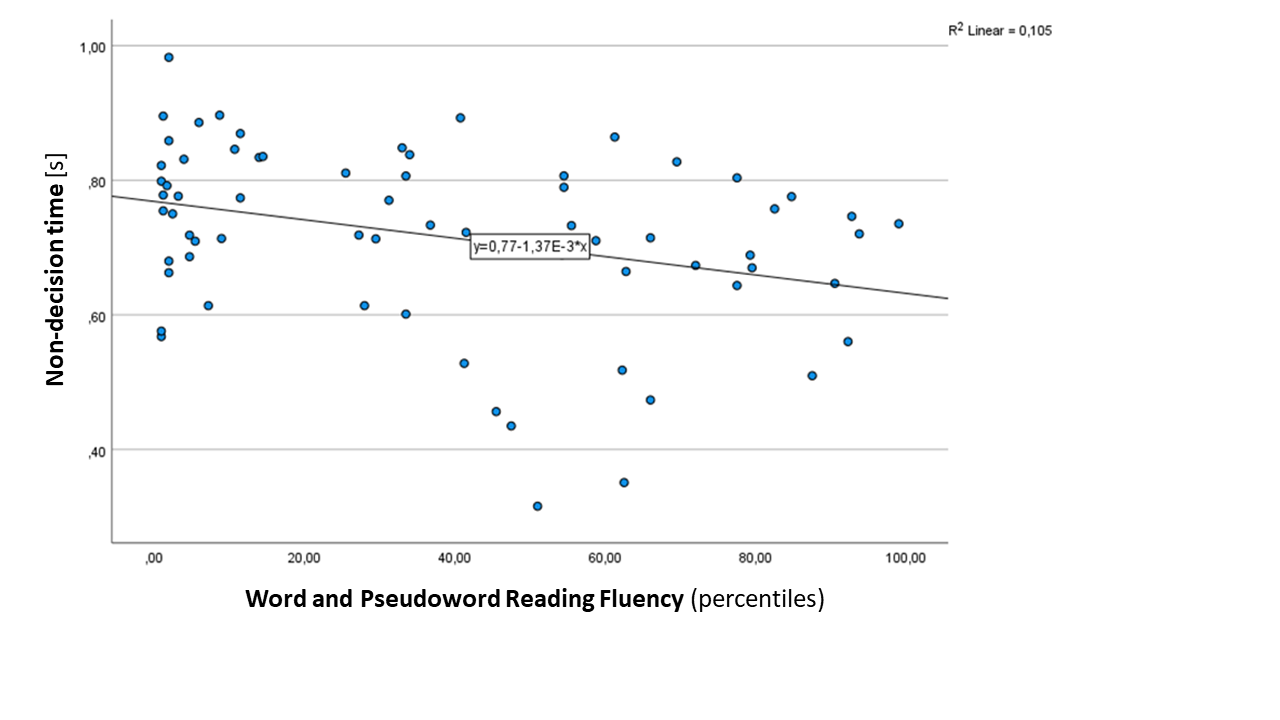

Supplement: Figure 3-2 — Non-decision time correlated with reading fluency (mean of pseudoword and word reading percentiles SLRT-II). (SLRT-II, r = -0.344, p < 0.001) (Figure 1-2). Download Figure 3-2, TIF file. [file jneuro-45-e1119242025-s012.tif]

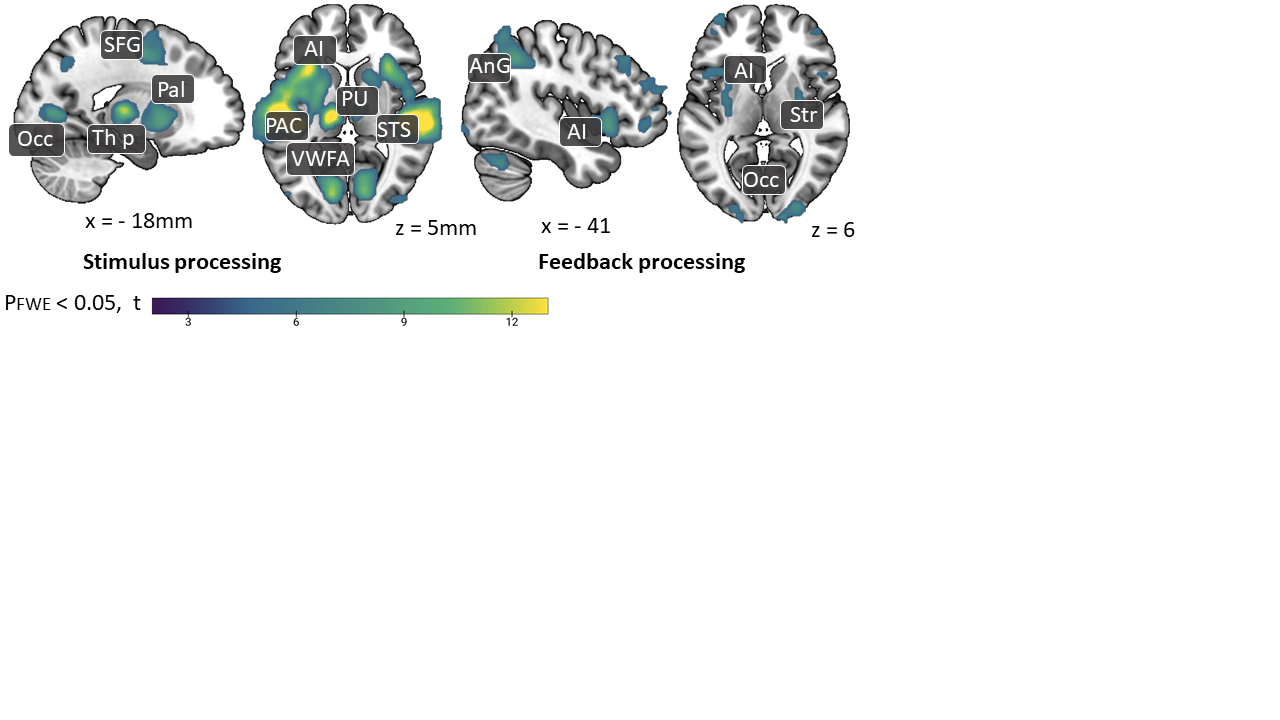

Supplement: Figure 4-1 — Conventional analyses; Left: brain activation at stimulus presentation: bilateral transverse temporal gyri, bilateral precentral gyri, bilateral putamen, thalamus, pallidum, superior frontal gyrus, superior temporal gyrus, anterior insula, bilateral middle frontal gyri, calcarine cortex, inferior and superior occipital. Right: brain activation during feedback processing: bilateral angular gyri, bilateral striatum (putamen, caudate), bilateral anterior insulae, bilateral middle frontal gyri, bilateral middle temporal gyri, bilateral superior frontal gyri, bilateral occipital poles/inferior occipital/lingual gyrus. The whole brain analyses of stimulus and feedback processing in the whole group of children (n = 80) indicated activation of an extended network processing the sensory information such as the bilateral auditory and visual regions, further in the anterior insulae (AI), putamen (PU), and superior parietal cortex, pre- and postcentral regions (PreC/PostC), anterior cingulate cortex (ACC) and parts of the right striatum (Str), during audio-visual stimulation. During feedback processing the bilateral angular gyri (AnG), striatal regions including the PU, AI, middle frontal gyri (MFG), and occipital poles were activated (Occ) (see Table 4-1). Download Figure 4-1, TIF file. [file jneuro-45-e1119242025-s013.tif]

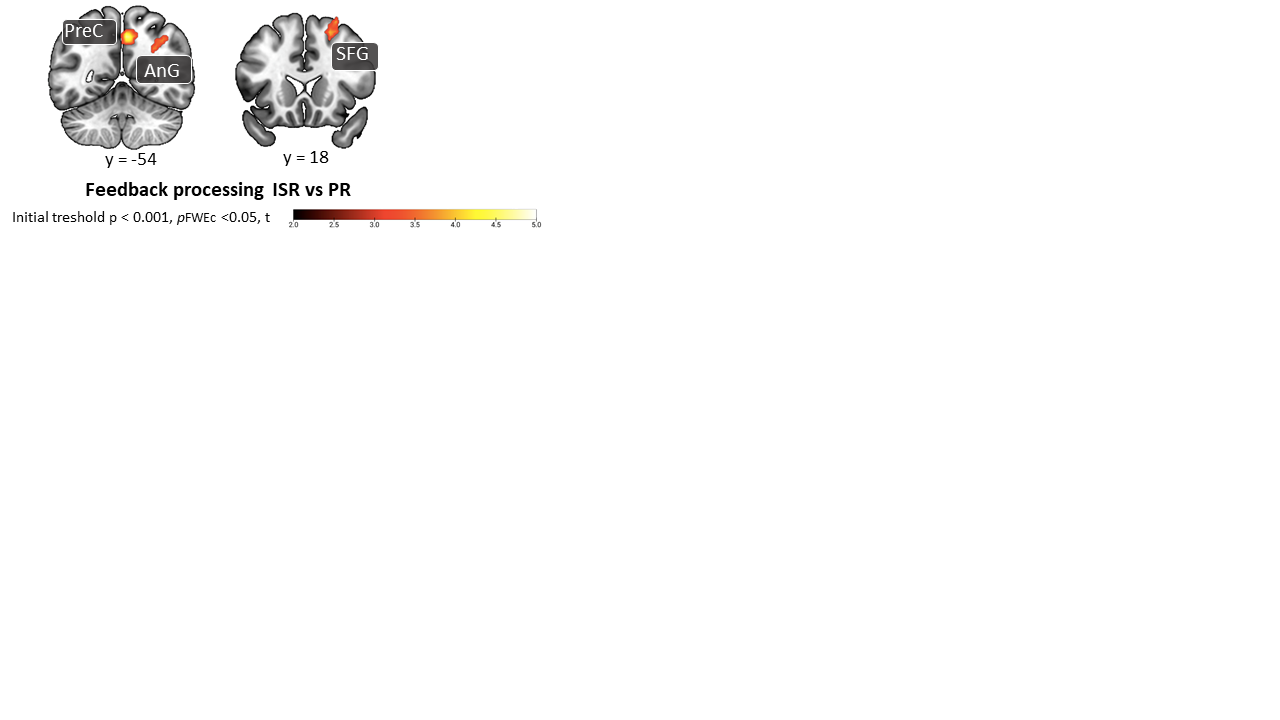

Supplement: Figure 4-2 — Group comparison of children with intermediate to strong (ISR, n = 42) vs poor reading (PR, n = 27) skills: feedback processing vs baseline; significant activation in right Precuneus, right Angular Gyrus, and right Superior Frontal Gyrus. Cluster defining threshold puncorr. = 0.001, cluster correction pFWEc < 0.05. Abbreviations: SFG = Superior Frontal Gyrus, AnG = Bilateral Angular Gyrus, PreC = Precentral Cortex. Additional group comparisons between children with intermediate to strong (n = 42) or poor reading skills (n = 27) yielded differences during feedback processing in the right angular gyrus (AnG), the precentral gyrus (PreC), and the superior frontal gyrus (SFG) (Table 4-2) but none during stimulus processing. Download Figure 4-2, TIF file. [file jneuro-45-e1119242025-s014.tif]

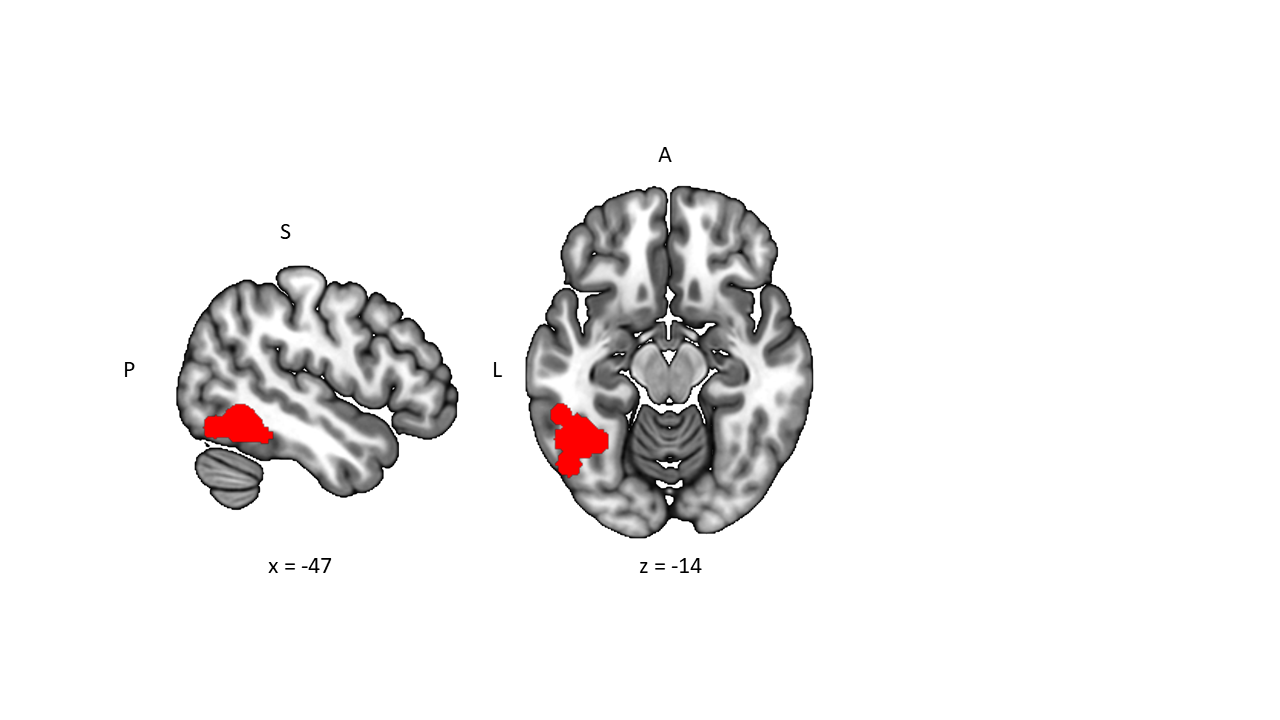

Supplement: Figure 4-3 — Literature-based mask of the Visual Word Form Area. Additional region of interest (ROI) analysis of the VWFA was performed to examine whether children with typical and with poor reading skills show differences in the visual processing of the false font characters during the LSS task. A literature-based VWFA mask was used (c.f. (Haugg et al. 2023)) which was created by defining spheres with different radii around the activation peaks reported in several articles on VWFA listed below using the MarsBaR toolbox for SPM (MARSBAR V0.41, http://marsbar.sourceforge.net/). These spherical ROIs were then combined to form a joint VWFA mask (see Table 4-3). Download Figure 4-3, TIF file. [file jneuro-45-e1119242025-s015.tif]

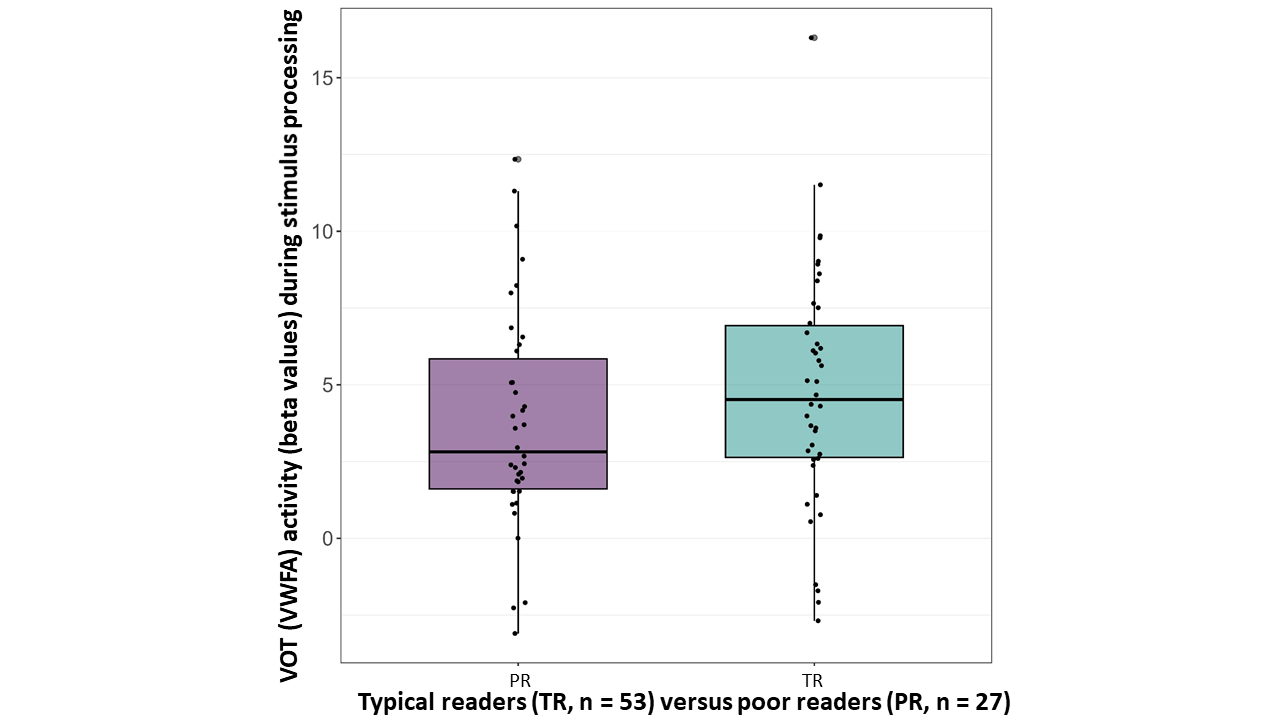

Supplement: Figure 4-4 — Group differences on a trend level between typical and poor readers. From the joint VWFA ROI, we extracted beta values using MarsBaR and conducted a two-sample t-test to compare activation in children with typical versus poor reading skills. There was no significant difference between groups in the activation of the VWFA during the LSS task. However, a statistical trend indicated that children with typical reading skills had marginally higher BOLD signal in the VWFA than children with poor reading skills (Figure 1-7). This was similar for the core sample of 80 children (t(78) = -1.709, p = 0.091) as well as after excluding the 11 children whose reading skills fell within the 16th to 25th percentile (t(67) = -0.670, p = 0.051). Download Figure 4-4, TIF file. [file jneuro-45-e1119242025-s020.tif]

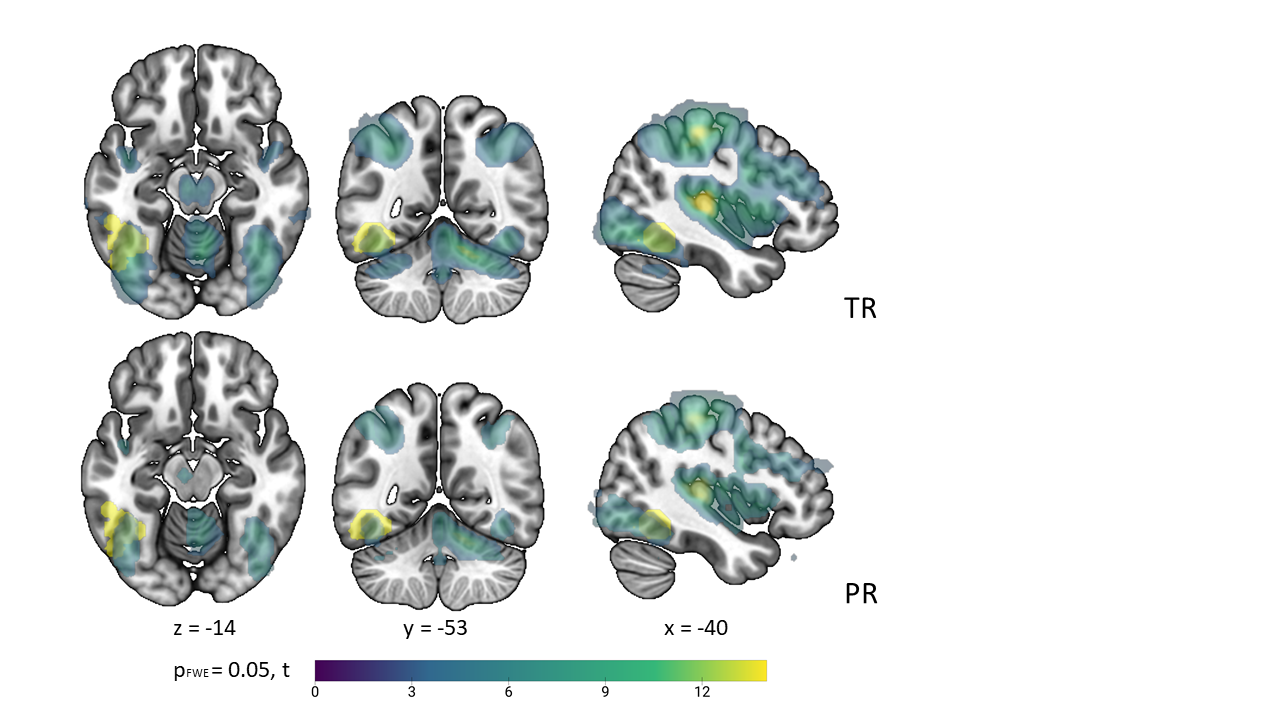

Supplement: Figure 4-5 — VWFA activation in children with typical and with poor reading skills. VWFA ROI in yellow, TR = children with typical reading skills (n = 53), PR = children with poor reading skills (n = 27). Download Figure 4-5, TIF file. [file jneuro-45-e1119242025-s021.tif]

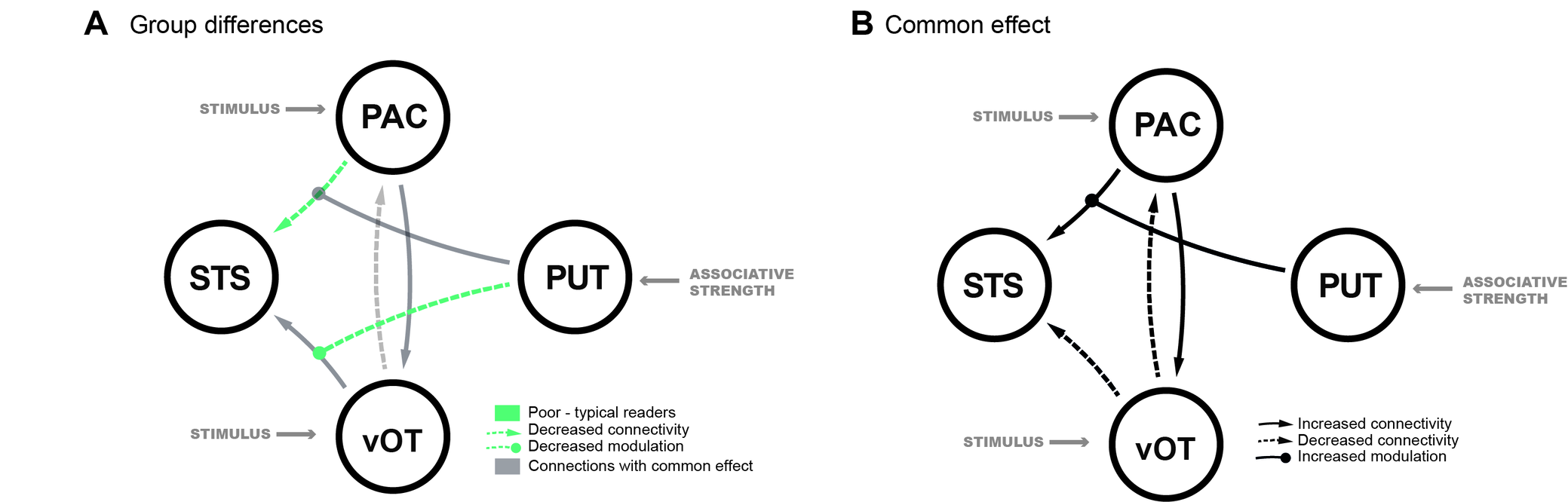

Supplement: Figure 5-1 — A Group difference in connectivity between children with intermediate to strong (ISR) and with poor reading skills. Gray lines represent baseline connectivity patterns observed across all children, while green lines indicate significant deviations in connectivity specific to the PR group. Decreased connectivity and modulations are shown as dashed lines. B. Common effects in effective connectivity across the whole group, regardless of reading skills. Solid lines represent positive connectivity and modulation, dashed lines show inhibitory connectivity. Download Figure 5-1, TIF file. [file jneuro-45-e1119242025-s016.tif]

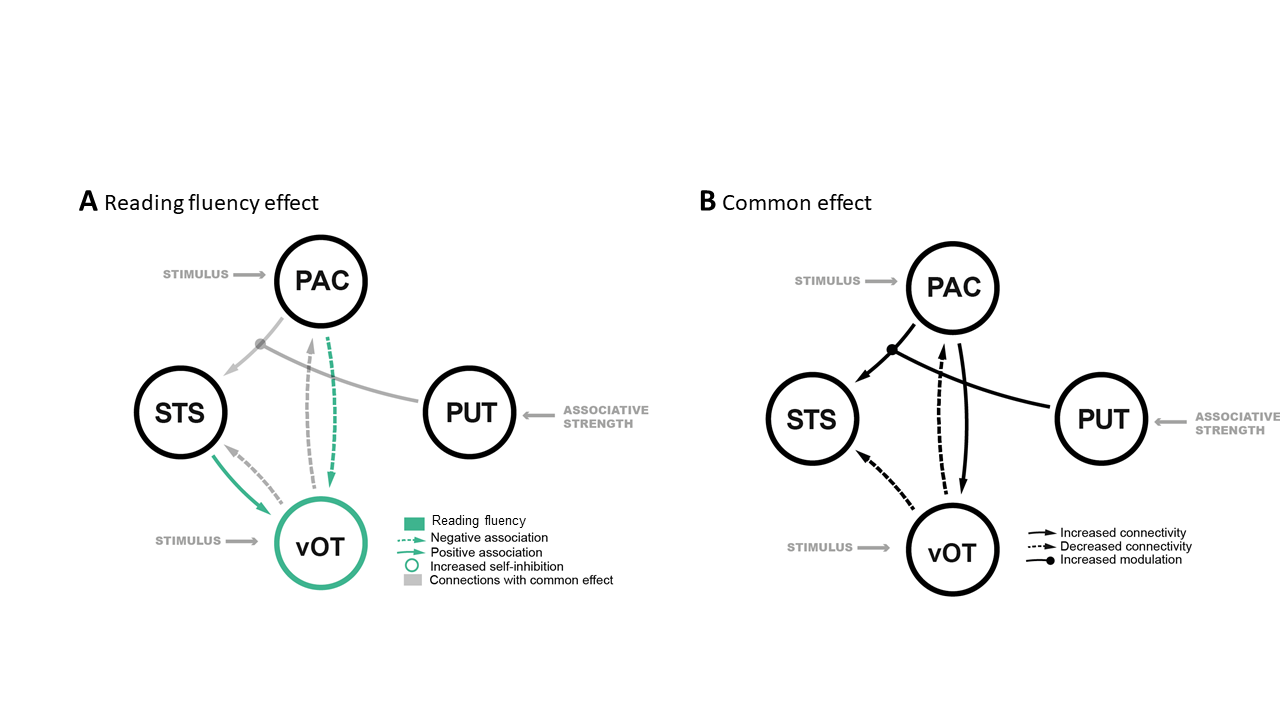

Supplement: Figure 5-2 — DCM with reading sore as a continuous variable. A. Effect of reading skills in the whole group. B. Common effects in the whole group (n = 75). We replicated our DCM analyses with the whole (n = 75) group of children and with reading score as a continuous variable. Overall, similar common effects were found when repeating the analysis with the reading fluency scores (instead of group assignments) as a predictor. We found a significant effect of reading ability on the vOT’s afferent connections and its self-connection (i.e. the input sensitivity of the region). Lower reading fluency scores were related to decreased self-inhibition of the vOT and connectivity from STS to vOT, and increased connectivity between PAC and vOT (Figure 1-10). In this model, the effect of the reading fluency score on the striatal modulation did not reach statistical significance (Table 5-2). Download Figure 5-2, TIF file. [file jneuro-45-e1119242025-s017.tif]
